# Erythroid Atypical Chemokine Receptor 1 Deficiency Aggravates Immune-Mediated Kidney Disease

**DOI:** 10.1681/ASN.0000000878

**Published:** 2025-09-17

**Authors:** Katharina Artinger, Igor Novitzky-Basso, Konstantin A. Klötzer, Nathaly Anto-Michel, Corinna Schabhüttl, Julia Christine Gutjahr, Maryna Samus, Elin Hub, Alexander H. Kirsch, Daniel Leitinger, Stefan Wernitznig, Theophilus Umeizudike, Marion Pollheimer, Maria H. Ulvmar, Agnes A. Mooslechner, Andrea Bacon, Philipp Eller, Thomas Kroneis, Dagmar Kratky, Alexander R. Rosenkranz, Kathrin Eller, Antal Rot

**Affiliations:** 1 Clinical Division of Nephrology, Medical University of Graz, Graz, Austria; 2 Faculty of Medicine and Dentistry, William Harvey Research Institute, Queen Mary University of London, London, United Kingdom; 3 Hans Messner Allogeneic Blood and Marrow Transplant Unit, Princess Margaret Cancer Centre, Toronto, Ontario, Canada; 4 Department of Medicine, University of Toronto, Toronto, Ontario, Canada; 5 Clinical Division of Cardiology, Medical University of Graz, Graz, Austria; 6 Institute of Cell Biology and Immunology Thurgau (BITG), University of Konstanz, Kreuzlingen, Switzerland; 7 Division of Cell Biology, Histology and Embryology, Gottfried Schatz Research Center, Medical University of Graz, Graz, Austria; 8 Nephrology Unit, Medicine Department, Lagos State University Teaching Hospital, Lagos, Nigeria; 9 Institute of Pathology, Medical University of Graz, Graz, Austria; 10 Department of Medical Biochemistry and Microbiology, Uppsala University, Uppsala, Sweden; 11 Division of Pharmacology, Otto Loewi Research Center, Medical University of Graz, Graz, Austria; 12 Institute of Immunology and Immunotherapy, University of Birmingham, Edgbaston, United Kingdom; 13 Intensive Care Unit, Department of Internal Medicine, Medical University of Graz, Graz, Austria; 14 Gottfried Schatz Research Center, Molecular Biology and Biochemistry, Medical University of Graz, Graz, Austria; 15 BioTechMed-Graz, Graz, Austria

**Keywords:** GN, immune complexes

## Abstract

**Key Points:**

- Selective erythroid atypical chemokine receptor 1 deficiency exacerbated disease activity in experimental GN *via* changes of the myeloid lineage.
- Activated monocytes and profibrogenic phenotypes of macrophages underlaid the kidney phenotype observed in erythroid-silent individuals.
- Genetically modified mouse models showed pathomechanisms of kidney diseases in patients of West African ancestry with erythroid atypical chemokine receptor 1 deficiency.

**Background:**

Single-nucleotide polymorphisms of the atypical chemokine receptor 1 (*ACKR1*) gene encode human Duffy antigen blood groups. Most individuals of West African ancestry carry a single-nucleotide polymorphism in the promoter region of the *ACKR1* gene that disrupts its transcription in erythroid cells but not in venular endothelial cells, leading to an erythroid-silent, FyBES Duffy phenotype.

**Methods:**

We used two mouse models of erythroid-selective ACKR1 deficiency to delineate the fundamental role of this receptor in the erythroid compartment in regulating the development of experimental immune-mediated kidney disease.

**Results:**

Humanized transgenic Duffy erythroid-silent Duffy-negative transgene mice and chimeric wild-type mice transplanted with ACKR1-deficient bone marrow, both selectively lacking erythroid ACKR1, showed increased disease activity and fibrosis after induction of nephrotoxic serum nephritis, as compared with their respective controls. Mice lacking erythroid ACKR1 exhibited altered serum chemokine levels and bone marrow monocytes displaying activated and promigratory phenotypes. Moreover, they showed an increase in kidney-infiltrating macrophages that were characterized by a profibrotic transcriptome signature. No changes in ACKR1 expression in kidney vascular endothelial cells were seen in erythroid ACKR1-deficient mice with or without nephrotoxic serum nephritis.

**Conclusions:**

Our data demonstrates that erythroid-specific ACKR1 deficiency led to an increased infiltration of the kidney by macrophages with an altered profibrotic phenotype in nephrotoxic serum nephritis, resulting in aggravated kidney disease.

## Visual Abstract

**Figure:**
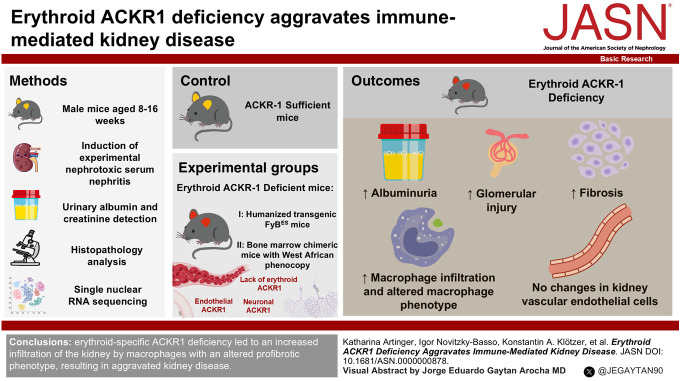

## Introduction

The greater susceptibility toward infections, such as coronavirus disease 2019,^1,2^ higher mortality in sepsis,^3^ and predisposition to the development of cancer^4^ are paradigmatic examples reflecting distinct, mostly unexplored patient group–specific pathomechanistic features that characterize diseases in individuals of African ancestry. West African individuals also have the highest frequency of CKD,^5–8^ further underscored by the heightened prevalence of certain kidney pathologies, including lupus nephritis.^9^ This traditionally has been attributed to combinations of confounding factors and concomitant comorbidities, such as salt sensitivity,^10^ hypertension,^11^ obesity,^12^ type 2 diabetes mellitus, and cardiovascular disease.^13^ The highest frequencies of apolipoprotein L1 (APOL1)–associated variants that confer risk of kidney disease are prevalent in individuals of West African ancestry.^14,15^ Nevertheless, a comprehensive understanding of the pathophysiologic mechanisms underpinning the higher susceptibility to kidney disease in this ethnic group remains elusive.

Atypical chemokine receptor 1 (ACKR1) was originally identified as the Duffy blood group antigen on erythrocytes^16^ and has subsequently been described on venular endothelium^17,18^ and cerebellar Purkinje neurons^19^ but not in immune or other cells. The alleged ACKR1 expression in monocytes and macrophages was due to an artifactual immunoreactivity of entirely nonspecific commercial antibodies and has been convincingly refuted.^20^ Two alternative codominant alleles of *ACKR1*, *FY×A*, and *FY×B*, which differ by a single nucleotide within the coding region, determine the two main Duffy blood groups FyA and Duffy B (FyB). Most individuals of West African ancestry carry a polymorphic *FY×B ACKR1* allele with a single-nucleotide polymorphism (rs2814778) within the promoter region of the erythroid transcription factor GATA binding protein 1. This disrupts GATA binding protein 1 binding and silences the transcription of *ACKR1* selectively in the erythroid cell lineage, thus causing the Duffy-negative erythroid-silent Duffy-negative (FyB^ES^) phenotype.^21,22^ Similar to the survival advantages provided by the polymorphic *APOL1* variants in *Trypanosoma brucei*–caused sleeping sickness,^23^ FyB^ES^ confers resistance toward malaria caused by *Plasmodium vivax.*^24^ This suggests a potential evolutionary selection mechanism and an almost 100% fixation of this single-nucleotide polymorphism in Western African populations.^21^

ACKR1 is a member of the atypical chemokine receptor family and can bind chemokines of the C-C motif and C-X-C motif families.^25–27^ It bears structural resemblance to G protein–coupled receptors (GPCRs); however, ACKR1 diverges fundamentally by lacking several functional GPCR domains, most notably the Asp(D)-Arg(R)-Tyr(Y)-Leu(L)-Ala(A)-Ile(I)-Val(V) consensus sequence in the second intracellular loop that is required for the binding of G-proteins. Accordingly, on chemokine ligation, ACKR1 does not trigger any of the conventional GPCR signaling pathways but binds and potentially internalizes chemokines. ACKR1 expression on the vascular endothelium of blood vessels increases leukocyte migration *via* chemokine transcytosis.^28,29^ On circulating red blood cells, ACKR1 acts as a chemokine buffer, whereby the sink or reservoir function is prioritized, depending on the particular chemokine and context.^30–32^ Furthermore, the absence of ACKR1 expression on nucleated erythroid cells in the bone marrow alters hematopoiesis and leads to the development of benign neutropenia.^33^

ACKR1 expression in the venular endothelial cells (ECs) and its lack on erythroid cells have previously been implicated in the pathogenesis of kidney disease and transplantation. On the one hand, it was noted that the number of renal interstitial venules expressing ACKR1 increased in human kidney biopsy samples of crescentic GN and transplant rejection.^34,35^ On the other hand, kidney allograft survival was decreased in individuals of West African ancestry who lack erythroid ACKR1 expression.^36^ In murine models, global *Ackr1* deficiency protected from acute renal ischemia-reperfusion injury,^37^ but disease parameters are aggravated in models of nephritis and unilateral ureteral obstruction.^38^ Experimental nephrotoxic serum nephritis is a mouse model reflecting several disease features of human immune complex GN that is characterized by a rapid loss of kidney function. The pathogenesis in this model is dependent on Th1 and Th17 cells as well as neutrophils and macrophages.^39–42^ This study aimed to investigate the contribution of erythroid ACKR1 to the pathomechanisms of immune-mediated kidney disease, using the experimental model of nephrotoxic serum nephritis.

## Methods

### Mouse Models

Male mice aged 8- to 16-weeks were used for all experiments. All mice were bred in-house and maintained in a virus-free environment at the Medical University of Graz. ACKR1-deficient mice (*ACKR1*^−/−^, *Ackr1*^tm1Scp^), originally described by Dawson *et al.*,^43^ were used for experimental procedures. Genotyping of wild-type (WT) and ACKR1-deficient mice was performed by PCR as previously described^43^ using the following primers to amplify a 400- and a 300-bp PCR product of the WT allele and the targeted *Ackr1* gene, respectively: primer1: 5′-GCT​AGA​TGT​CCT​GAC​TGT​CC; primer2: 5′-CCA​GTA​GCC​CAG​GTT​GCA​TA; primer3: 5′-TAT​GGC​GCG​CCA​TCG​ATC​TC-3. Bone marrow chimeric mice were produced by transplantation of C57BL/6J (WT) and ACKR1-deficient bone marrow into WT mice as detailed in Supplemental Methods. Humanized erythroid ACKR1-deficient and their control ACKR1-sufficient mice transgenic for human single-nucleotide polymorphisms rs2814778 (causing FyB^ES^) and rs12075 (causing FyB) were generated as detailed in Supplemental Methods and are designated here as FyB^ES^ transgene (FyB^ES^TG) and FyB transgene (FyBTG), respectively (Supplemental Figure 1). The transgenesis was performed as described previously,^44^ with a key difference as detailed in Supplemental Methods.

### Induction of Experimental Nephrotoxic Serum Nephritis

Nephrotoxic serum nephritis was induced as described previously.^45^ Mice were group-housed in individually ventilated cages with a 12-hour light–dark cycle. Briefly, (*1*) ACKR1-deficient and WT, (*2*) FyB^ES^TG and FyBTG, and (*3*) erythroid ACKR1-deficient and -sufficient chimeric mice were preimmunized subcutaneous with 2 mg/ml rabbit IgG (Jackson ImmunoResearch Laboratories, West Grove, PA) dissolved in incomplete Freund's adjuvant (Sigma-Aldrich, St. Louis, MO) supplemented with desiccated, nonviable *Mycobacterium tuberculosis* H37a (Difco Laboratories, Detroit, MI). Three days later, 100 *µ*l nephrotoxic serum from rabbits immunized with mouse glomerular basement membrane was injected intravenously *via* the tail vein. The nephrotoxic serum was prepared as described previously.^46,47^

### Single-Nuclei RNA-Sequencing and Data Integration

Cell nuclei were isolated from nephritic kidneys of erythroid-specific ACKR1-deficient and -sufficient chimeric mice (*n*=2/group) using the Chromium Nuclei Isolation Kit (10x Genomics, Pleasanton, CA). Single-nuclei and gel bead emulsion were generated from isolated nuclei on a Chromium Controller (10x Genomics).^48^ Single-nuclei RNA-sequencing (snRNAseq) libraries were prepared using the Chromium Next Gel Beads-in-emulsion Single Cell 3′ Kit v3.1 (10x Genomics). Samples were sequenced on an Illumina NovaSeq sequencer using paired-end sequencing (Vienna BioCenter Core Facility, Vienna, Austria). Sequencing data were processed using the Cell Ranger Single Cell software suite 7.0.1 (10x Genomics).^48^ Further data processing and snRNAseq analysis were performed as detailed in Supplemental Methods.^49–51^

### Urinary Albumin and Urinary Creatinine Detection

A double-sandwich ELISA (Abcam, Cambridge, MA) was used for the determination of urinary albumin excretion as reported previously.^40^ Urinary creatinine was evaluated photometrically using a commercially available picric acid–based kit (Sigma-Aldrich).

### Immunofluorescence and Confocal Microscopy

For immunofluorescence staining, tissue and cells grown in chamber slides were fixed either in ice-cold acetone or in 2% paraformaldehyde and stained as detailed in Supplemental Methods. The stainings were evaluated using LSM710 (Zeiss, Oberkochen, Germany) or Olympus FV3000 (Olympus, Shinjuku, Japan) confocal microscopes.

### Histopathology

For periodic acid–Schiff (PAS) and Picro-Sirius red staining, formalin-fixed kidney tissue was embedded in paraffin and cut into 4-*µ*m sections before staining. Evaluation was performed as detailed in Supplemental Methods.

### Reverse Transcription Real-Time PCR

RNA extraction, reverse transcription of RNA, and real-time PCR were performed as detailed in Supplemental Methods.

### Flow Cytometry

Stainings of erythrocytes from whole blood and leukocyte populations from lymphoid tissue, kidneys,^52^ bone marrow, and blood were performed as detailed in Supplemental Methods. The samples were analyzed on a CytoFLEX LX cytometer (Beckman Coulter, Brea, CA) or a FACS Calibur flow cytometer (BD Biosciences).

### Isolation, Differentiation, and Polarization of Bone Marrow–Derived Macrophages

Bone marrow was isolated from WT and *Ackr1*^−/−^ mice and cultured in chamber slides or 12-well plates in DMEM with 10% FCS, 1% penicillin/streptomycin, and 20 ng/ml macrophage colony-stimulating factor. After 7 days, the cells were polarized with 100 ng/ml LPS or 20 ng/ml IL-4 for 24 hours, and cells were quantified for their positivity of F4/80 and Arg-1 with flow cytometry and immunofluorescent stainings on an Olympus FV3000 Laser scanning microscope (Olympus) using QuPath.

### Phagocytosis Assay

Bone marrow–derived macrophages were incubated with pHrodo Green *Staphylococcus aureus* BioParticles Conjugate for Phagocytosis (Invitrogen, Waltham, MA) at 37°C. After 1 hour, the samples were placed on ice and stained for cell surface markers before phagocytosis of *Staphylococcus aureus* was evaluated on a CytoFLEX SI cytometer (Beckman Coulter).

### Evaluation of Cytokines and Chemokines in Cell Culture Supernatant and Serum

Multiplex immunoassays were performed according to the manufacturer's instructions. We used LEGENDplex MU Proinflammatory Chemokine Panel and Proinflammatory Chemokine Panel 2 (Biolegend, San Diego, CA) for the detection of ACKR1-binding chemokines and LEGENDplex MU Macrophage/Microglia Panel (Biolegend) for the analysis of chemokines in the supernatant of bone marrow–derived macrophages.

### Study Approvals

All animal experiments were approved by the Animal Welfare Committee of Austria (BMWFW-66.010/0074-WF/V/3b/2017 and BMWFW-66.010/0054-WF/V/eb/2015). All animal experiments were conducted with strict adherence to the laws of Austria (BGBl. I Nr. 118/2004).

Human blood was taken from healthy volunteers following the ethical approval by the Ethics Committee of the School of Medical and Dental Sciences, University of Birmingham, United Kingdom, following informed consent. Archived frozen blocks of healthy human spleen were obtained from the Human Biomaterials Resource Centre at the University of Birmingham and used in accordance with the ethical approval by the Ethics Committee of the School of Medical and Dental Sciences, University of Birmingham.

### Statistical Analysis

All statistical evaluations were performed using GraphPad Prism 6.0 (GraphPad Software, La Jolla, CA), and the results are shown as means with SEM. Testing for normality was done using the Kolmogorov–Smirnov test with Dallal–Wilkinson–Lillifors correction and the Shapiro–Wilk normality test. According to the distribution, an unpaired *t* test or Mann–Whitney test was used. When three or more groups were compared, ANOVA or Kruskal–Wallis test and *post hoc* tests were performed. A *P* value < 0.05 was considered statistically significant. Justification of sample size: For the experiments presented in bone marrow chimeric mice and transgenic humanized mice, a total of nine and seven animals per experimental group were used, respectively, across three and two independent experiments. The reported *n* values for *in vivo* studies represent pooled data from these independent experiments. Within-group variability in independent experiments was quantified using the SD and coefficient of variation. Minor discrepancies in *n* values across individual graphs are due to limitations in sample quality or availability, as well as occasional experimental dropouts. Minimum detectable effect sensitivity analyses were conducted using G×Power^53^ (*α*=0.05, two-tailed, 80% power). These analyses determined the smallest effect sizes that our sample sizes could reliably detect. Effect sizes were initially expressed as Cohen d and subsequently corrected to Hedges g to account for small-sample bias. With *n*=9 in ACKR1-deficient and -sufficient mice and *n*=7 in transgenic humanized mice, the study had 80% power to detect an effect size of at least Hedges g≈1.33 and Hedges g≈1.52, respectively. With *n*=6 and *n*=5 per group, the study had 80% power to detect an effect size of at least Hedges g≈1.65 and Hedges g≈1.81, respectively. Smaller effects may not have been detectable with the presented sample size. The overall sample size was constrained by the exploratory nature of this study, which constitutes the first *in vivo* evaluation of bone marrow chimeric mice with erythroid-selective ACKR1 deficiency as well as FyBTG and FyB^ES^TG mouse models.

**Figure 1:**
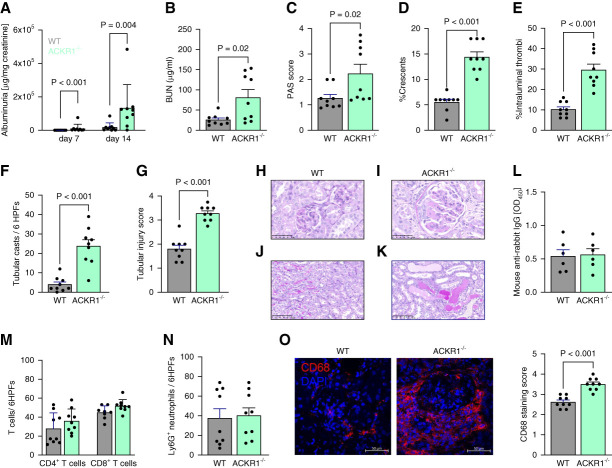
**Global ACKR1 deficiency aggravates nephrotoxic serum nephritis.** (A) Urinary albumin/creatinine ratio in WT and *Ackr1*^−/−^ mice 7 and 14 days after nephrotoxic serum nephritis induction (gray and green, respectively, and hereafter). (B) BUN in WT and *Ackr1*^−/−^ mice 14 days after nephrotoxic serum nephritis induction. (C) Glomerular PAS score, (D) glomerular crescents, (E) capillary intraluminal thrombi, (F) tubular casts, and (G) tubular injury score in WT and *Ackr1*^−/−^ mice 14 days after nephrotoxic serum nephritis. (H–K) Representative images of PAS-stained glomeruli and tubuli from (H and J) WT and (I and K) *Ackr1*^−/−^ mice 14 days after induction of nephrotoxic serum nephritis. (L) Mouse anti-rabbit IgG serum concentration in nephritic WT and *Ackr1*^−/−^ mice measured 14 days after induction of nephrotoxic serum nephritis at OD450. 1:64,000 serum dilution is shown. (M) CD4^+^ and CD8^+^ T cells and (N) Ly6G^+^ neutrophils counts in six renal HPFs/mouse. (O) CD68^+^ macrophages in kidneys of nephritic WT and *Ackr1*^−/−^ mice 14 days after induction of nephrotoxic serum nephritis. Representative micrographs of renal infiltrates stained with anti-CD68 (macrophages, red) and DAPI (nuclei, blue) and their scores. *n*=9 (A–G, M, N, and O) and *n*=6 (L) in each group from two independent experiments. (H, I, and O) Scale bars, 50 *µ*m. (J and K) Scale bars, 100 *µ*m. All data are presented as mean+SEM. *ACKR1*, atypical chemokine receptor 1; DAPI, 4′,6-diamidino-2-phenylindole; HPF, high-power field; PAS, periodic acid–Schiff; WT, wild-type.

**Figure 2:**
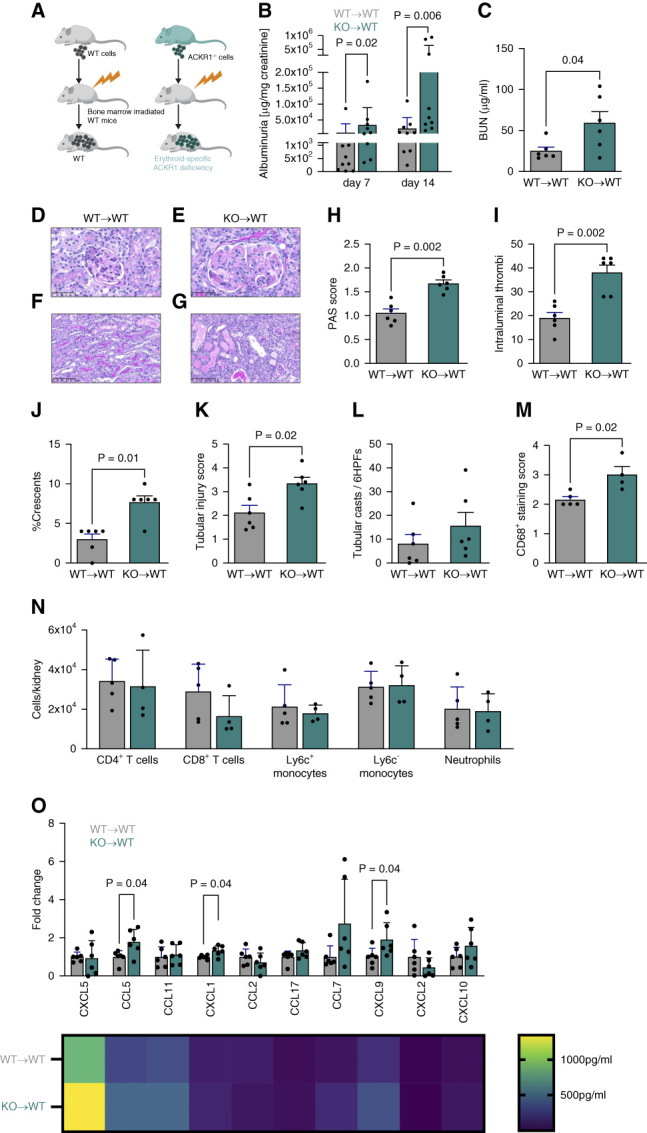
**Aggravated phenotype of nephrotoxic serum nephritis in bone marrow chimeric mice with the erythroid-selective *ACKR1* deficiency.** (A) Schematic representation of the generation of chimeric mice by bone marrow transplantation. WT mice were lethally irradiated and subsequently reconstituted with bone marrow cells from WT (gray) or *Ackr1*^−/−^ mice (turquoise, *i.e*., erythroid-selective *Ackr1*^−/−^ mice). (B) Urinary albumin/creatinine ratio in WT mice reconstituted with WT or *Ackr1*^−/−^ cells 7 and 14 days after nephrotoxic serum nephritis induction. (C) BUN 14 days after nephrotoxic serum nephritis induction in WT mice reconstituted with WT or *Ackr1*^−/−^ cells. (D–G) Representative PAS-stained micrographs of irradiated WT mice reconstituted with (D and F) WT (WT→WT) or (E and G) *Ackr1*^−/−^ bone marrow (KO→WT). (H) PAS scores, (I) capillary intraluminal thrombi, (J) crescent formation, (K) tubular injury, and (L) tubular casts in the mice 14 days after nephrotoxic serum nephritis induction. Kidney-infiltrating macrophages (M) and T cells, monocytes, and neutrophils (N) in the mice 14 days after nephrotoxic serum nephritis induction. (O) Serum chemokine levels in the mice 14 days after nephrotoxic serum nephritis induction. (D and E) Scale bars, 50 *µ*m. (F and G) Scale bars, 100 *µ*m. *n*=9 in each group (B) and *n*=6 in each group (C, H–L, and O); *n*=5 in mice reconstituted with bone marrow cells from WT mice (M and N) and *n*=4 (M and N) in mice reconstituted with bone marrow cells from *Ackr1*^−/−^ mice (due to drop-outs) from two independent experiments. All data show mean+SEM. KO, knockout.

**Figure 3:**
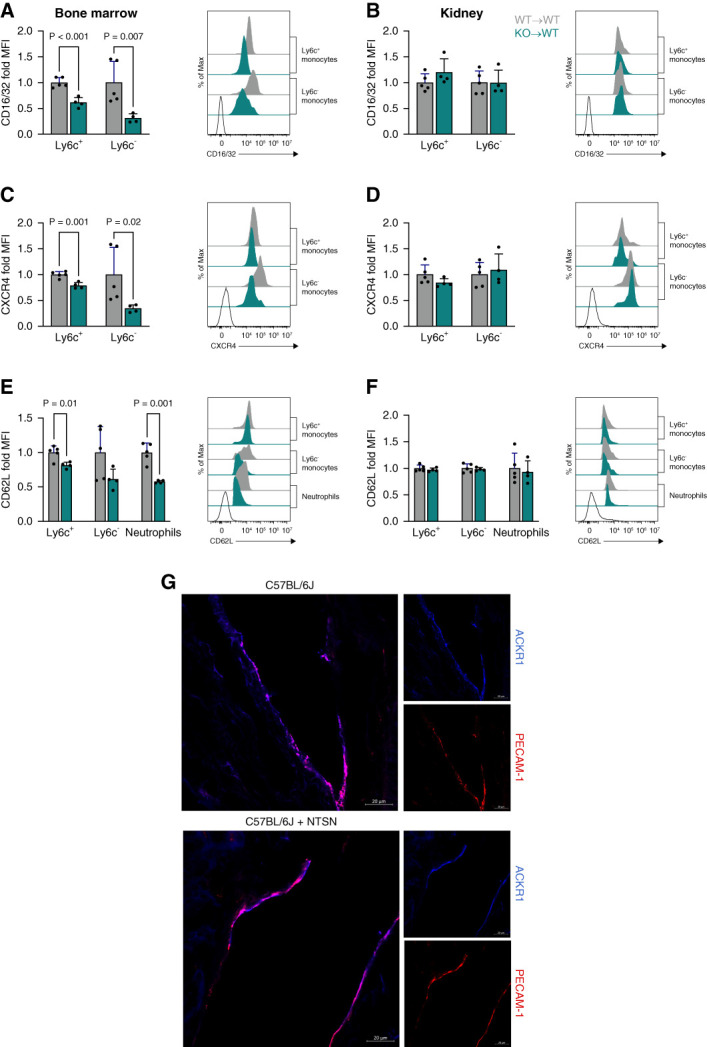
**Altered leukocyte phenotypes in nephritic bone marrow chimeric mice with the erythroid-selective ACKR1 deficiency.** Leukocyte phenotypes in irradiated WT mice reconstituted with WT or *Ackr1*^−/−^ bone marrow cells (*i.e*., erythroid-selective ACKR1-deficient mice) 14 days after nephrotoxic serum nephritis induction. CD16/32 expression on (A) bone marrow and (B) kidney-infiltrating Ly6c^+^ and Ly6c^−^ monocytes. CXCR4 expression on (C) bone marrow and (D) kidney-infiltrating Ly6c^+^ and Ly6c^−^ monocytes. CD62L expression on (E) bone marrow and (F) kidney-infiltrating Ly6c^+^ and Ly6c^−^ monocytes and neutrophils. *n*=5 in mice reconstituted with bone marrow cells from WT mice and *n*=4 in mice reconstituted with bone marrow cells from ACKR1^−/−^ mice. (G) Immunofluorescence micrographs of healthy WT kidney and WT kidney after 14 days of nephrotoxic serum nephritis stained with anti-ACKR1 (blue) and anti-PECAM-1 (red). Scale bars, 20 *µ*m. All data show mean+SEM. CXCR4, C-X-C motif chemokine receptor 4; MFI, median fluorescence intensity; PECAM-1, platelet and endothelial cell adhesion molecule 1.

## Results

### Erythroid-Specific Deletion of ACKR1 Aggravated Immune-Mediated Kidney Disease

To explore if ACKR1 plays a role in the pathomechanism of inflammatory kidney disease, we compared parameters of nephrotoxic serum nephritis in mice with global ACKR1 deficiency versus WT controls. ACKR1-deficient mice had significantly more severe glomerular injury, reflected in increased albuminuria (Figure 1A), elevated BUN (Figure 1B), PAS-positive glomerular deposits (Figure 1C), glomerular crescent formation, and capillary intraluminal thrombi (Figure 1, D, E, H, and I, and Supplemental Figure 2). Furthermore, tubular injury was more pronounced (Figure 1, F, G, J, and K, and Supplemental Figure 2). Despite the known limitations of picric acid–based assays for measuring urinary creatinine, the observed increase in albumin/creatinine ratio is thus supported by the concordant aggravation in other indicators of disease severity. Although no difference was detected in glomerular IgG deposition (Supplemental Table 1) and the humoral antibody response (Figure 3L and Supplemental Figure 2B), significantly more macrophages infiltrated the kidneys of ACKR1-deficient versus WT mice (Figure 1O and Supplemental Figure 2, C and D). Conversely, the numbers of kidney-infiltrating T cells and neutrophils were not significantly different between the two strains (Figure 1, M and N). Furthermore, the expression level of T-cell subset-specific markers in the kidney did not differ significantly between WT and ACKR1^−/−^ mice (Supplemental Figure 2E). Of note, when macrophages were depleted in nephritic ACKR1-deficient mice, nephrotoxic serum nephritis was ameliorated to the level observed in WT mice (Supplemental Figure 2, G–J).

In humans, an erythroid-selective ACKR1 deficiency associated with the Duffy-negative phenotype is commonly caused by the West African single-nucleotide polymorphism in the *ACKR1* promoter region, leading to a disproportional high incidence of kidney disease in individuals of West African ancestry. Therefore, we next explored the potential contribution of erythroid-selective ACKR1 deficiency to the severity of experimental nephrotoxic serum nephritis in mice. To this end, lethally irradiated WT mice were rescued by the reconstitution with either ACKR1-deficient or WT bone marrow,^54^ resulting in ACKR1-sufficient controls (WT→WT) and mice with an erythroid-selective ACKR1 deficiency (knock-out→WT), respectively (Figure 2A). The latter chimeric mice functionally have an erythroid-specific ACKR1 deficiency.^33^ This is due to a prominent selective expression of ACKR1 in the erythroid lineage and its well-documented absence in any of other hematopoietic lineages within the bone marrow.^20^ Two months after irradiation and bone marrow reconstitution, chimerism was confirmed by PCR from peripheral blood and flow cytometry of Ter-119^+^ erythroid cells from bone marrow (Supplemental Figure 3). In addition, flow cytometry confirmed the absence of ACKR1 on CD11b^+^ myeloid populations in the bone marrow of both ACKR1-deficient mice and irradiated WT mice reconstituted with ACKR1-deficient bone marrow and excluded changes in ACKR1 expression after induction of nephrotoxic serum nephritis (Supplemental Figure 3). Selective ACKR1 deficiency in the erythroid lineage caused an even more severe kidney disease phenotype as indicated by increased albuminuria (Figure 2B), increased BUN (Figure 2C), and more tissue damage as assessed by histology 14 days after nephrotoxic serum nephritis induction (Figure 2, D–L). Flow cytometry revealed that the numbers of T cells, monocytes, and neutrophils infiltrating the kidneys were comparable between the two groups (Figure 2N), whereas significantly more macrophages infiltrated the kidneys of mice with erythroid-selective ACKR1 deficiency (Figure 2M). Of note, we also analyzed T cells in draining lymph nodes but found no differences between the two groups in the numbers of neither CD4^+^ nor CD8^+^ cells (Supplemental Figure 4, A and B) or regulatory T cells (Supplemental Figure 4, C and D). In addition, serum concentrations of mouse anti-rabbit IgG antibody and its deposition on the glomerular basement membrane were unchanged (data not shown). In summary, mice with an erythroid-selective ACKR1 deficiency develop an aggravated phenotype of inflammatory kidney disease with an increase in renal macrophage infiltration as compared with controls, supporting the overarching hypothesis that ACKR1 expressed on erythroid cells protects from inflammatory kidney disease.

### Mice with Erythroid ACKR1 Deficiency Displayed an Altered Migratory Phenotype of Myeloid Cells in Nephrotoxic Serum Nephritis

On the one hand, erythroid ACKR1 plays a role as a chemokine buffer^4,27,31,32^; on the other hand, ACKR1 expressed on nucleated erythroid cells in the bone marrow regulates the development of the myeloid lineage,^33^ and both mechanisms might explain our observations. To assess the role of erythrocyte ACKR1 as a chemokine sink, we measured the abundance of ACKR1-cognate chemokines in peripheral blood of WT and erythroid ACKR1-deficient mice. However, only CCL5, CXCL1, and CXCL9 were significantly increased in the peripheral blood (Figure 2O) and are possibly not sufficient to fully explain the marked difference in the disease outcome of these mice. As myeloid cells are known drivers of the kidney disease in this model,^41^ we used flow cytometry (Supplemental Figure 5) to evaluate their phenotypes in the bone marrow (Figure 3, A, C, and E) and the kidney (Figure 3, B, D, and F). We recorded cell markers of activation and cytotoxicity effectors for both Ly6c^+^ and Ly6c^−^ monocytes, *i.e*., classical and nonclassical subpopulations, respectively. In erythroid-specific ACKR1-deficient mice, expression of CD16/32 and C-X-C motif chemokine receptor 4 (CXCR4) was decreased in both classical and nonclassical monocytes in the bone marrow (Figure 3, A and C), but unchanged in the kidney (Figure 3, B and D). Of note, CD16/32 was also decreased on monocytes and neutrophils in the peripheral blood of nephritic ACKR1-deficient mice as compared with WT controls (Supplemental Figure 2D). We also analyzed CD62L on classical and nonclassical monocytes as well as on neutrophils. All three populations had drastically lower expression levels of CD62L in the bone marrow (Figure 3E) but not in the kidney of mice with erythroid ACKR1 deficiency (Figure 3F). To investigate potential mechanisms involved in differential myeloid activation, we next isolated bone marrow from WT and global ACKR1-deficient mice and differentiated the cells to bone marrow–derived macrophages. Bone marrow–derived macrophages from ACKR1-deficient mice presented enhanced phagocytic capacity and significantly increased CD86 expression, whereas the expression levels of F4/80 remained comparable between the two groups (Supplemental Figure 6, A–D). Polarization in the presence of IL-4 led to increased arginase-1 expression in bone marrow–derived macrophages from ACKR1-deficient compared with WT mice (Supplemental Figure 6D). The polarized cells also tended to produce more IL-23, IL-12p40, IL-12p70, IL-1*β*, TNF-*α*, TGF-*β*1, CCL17, and granulocyte colony-stimulating factor (Supplemental Figure 6E).

**Figure 4:**
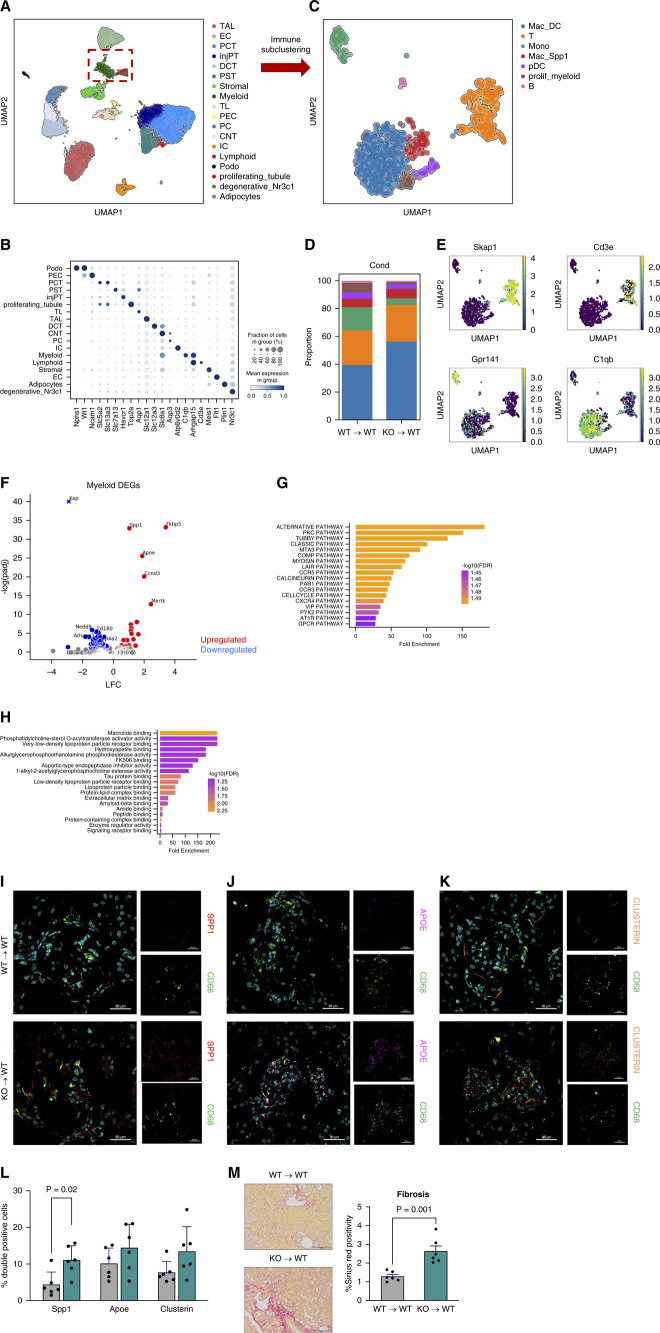
**Profibrotic macrophage phenotype in nephritic kidneys of erythroid-selective ACKR1-deficient mice.** (A) UMAP of kidney snRNAseq data of nephritic WT mice transplanted with WT and *Ackr1*^−/−^ bone marrow (*i.e*., erythroid-selective ACKR1-deficient mice). (B) Dot plot of representative marker genes used for annotation of cell clusters in the dataset. (C) Subclustering of immune cells in UMAP embedding. (D) Bar plot showing immune cell fractions with percentages of identified immune cell types in WT mice transplanted with WT (WT→WT) or *Ackr1*^−/−^ bone marrow (KO→WT). (E) Feature plots (UMAP embedding) visualizing the marker gene expression of immune cell subtypes, with the color scale maximum set to the 99th percentile of expression values. (F) Volcano plot of differentially expressed genes in myeloid cells relative to control. −log_10_ (adjusted *P* values) were capped at 40 for visualization purposes. (G and H) Pathway enrichment analysis of differentially expressed genes in myeloid cells of nephritic WT→WT and KO→WT in the kidney. Results from (G) gene ontology analysis molecular function and (H) biocarta were determined using ShinyGO 0.82. For each database, up to 20 pathways (FDR <0.1) are shown. Pathway enrichment analysis of upregulated genes was performed separately for each database. Coexpression of CD68 (macrophages, green) with (I) Spp1 (red), (J) ApoE (pink), and (K) clusterin (orange) using confocal microscopy, and (L) quantification of costainings. (M) Quantification of fibrosis and representative micrographs of Picro-Sirius red-stained nephritic kidney sections. (A–H) *n*=2 mice/group and (L and M) *n*=6/group. (I–K) Scale bars, 30 *µ*m. (M) Scale bars, 100 *µ*m. Data are presented as mean+SEM. CNT, connecting tubule; DCT, distal convoluted tubule; DEG, differentially expressed genes; EC, endothelial cell; FDR, false discovery rate; GPCR, G protein–coupled receptor; IC, intercalated cell; InjPT, injured proximal tubule; PC, principal cell; PCT, proximal convoluted tubule; pDC, plasmacytoic dendritic cells; PEC, parietal epithelial cell; PKC, protein kinase C; PST, proximal straight tubule; snRNAseq, single-nuclei RNA-sequencing; TAL, thick ascending limb; TL, thin limb; UMAP, Uniform Manifold Approximation and Projection; VIP, vasoactive intestinal peptide.

ACKR1 is expressed on venular ECs, where it mediates leukocyte transmigration.^17,18,28^ Next, we evaluated in both experimental strains any putative changes in the ACKR1 expression within the EC compartment, both in healthy mouse kidneys and those with nephrotoxic serum nephritis. To this end, we used a specific monoclonal anti-mouse ACKR1 antibody^17,20^ to visualize the expression of ACKR1 in frozen sections by confocal laser scanning microscopy. In all samples, ACKR1 immunoreactivity associated with renal venules and veins only, and no apparent changes in ACKR1 expression were observed after induction of nephrotoxic serum nephritis (Figure 3G). Glomerular capillary ECs did not express ACKR1, neither in healthy nor nephritic kidneys (Supplemental Figure 7).

### Profibrotic Phenotype in Macrophages Infiltrating Kidneys of Nephritic Mice with Erythroid-Selective ACKR1 Deficiency

To further characterize the macrophage population infiltrating the nephritic kidney, we determined their transcriptome. To this end, we performed snRNAseq from the kidneys of WT or erythroid-specific ACKR1-deficient mice 14 days after induction of nephrotoxic serum nephritis. Following quality control, 25,100 cells from WT and erythroid-specific ACKR1-deficient kidneys were analyzed. Unsupervised clustering revealed 18 distinct cell populations, distinguished by their unique marker gene expression (Figure 4, A and B).^55^ These cell clusters included a myeloid and lymphoid population, expressing *C1qb* and *Cd3e*, respectively (Figure 4B). To describe immune cells in our dataset in more detail, we next applied an iterative subclustering approach (Supplemental Methods). Within the immune cell cluster, seven distinct cell populations were identified, comprising macrophages/dendritic cells, T cells, monocytes, Spp1^+^ macrophages, plasmacytoic dendritic cells, proliferating myeloid cells, and B cells (Figure 4, C and D, and Supplemental Figure 8). Marker gene expression aligned with previously published single-cell kidney datasets^56^ and protein expression databases.^57^ Lineage-specific marker genes, such as *Skap1* and *CD3e* for T cells, and *Gpr141* and *C1qb* for myeloid cells, were used for classification (Figure 4E). Notably, we identified a macrophage subset with elevated *Spp1* expression (Supplemental Figure 8), which was designated as Spp1^+^ macrophages. Analysis of the renal immune cell composition confirmed an increased proportion of macrophages (Mac_DC population) in erythroid-specific ACKR1-deficient mice (Figure 4D) with no apparent changes in proportions of other cell types (Supplemental Figure 9). *Apoe*, *Spp1* (alias osteopontin), *Fkbp5*, *Ccnd3*, *clusterin*, and *Mertk* were among the most prominently upregulated genes in myeloid cells (Figure 4F and Supplemental Table 2). Pathway enrichment analysis (Figure 4, G and H) of differentially expressed genes in myeloid cells further elucidates the molecular characteristics of this macrophage phenotype. Notably, the analyses revealed significant enrichment of pathways related to complement activation, phagocytosis,^58,59^ fibrosis,^60^ macrophage polarization,^61,62^ and lipid metabolism—hallmarks previously associated with lipid-associated macrophages.^63^ We next verified increased ApoE, clusterin, and Spp1 protein expression in CD68^+^ infiltrating renal macrophages by immunofluorescence staining and quantification (Figure 4, I–L). Macrophages that express ApoE, clusterin, and/or Spp1 have been closely linked to the development of a profibrotic macrophage phenotype.^64–67^

**Figure 5:**
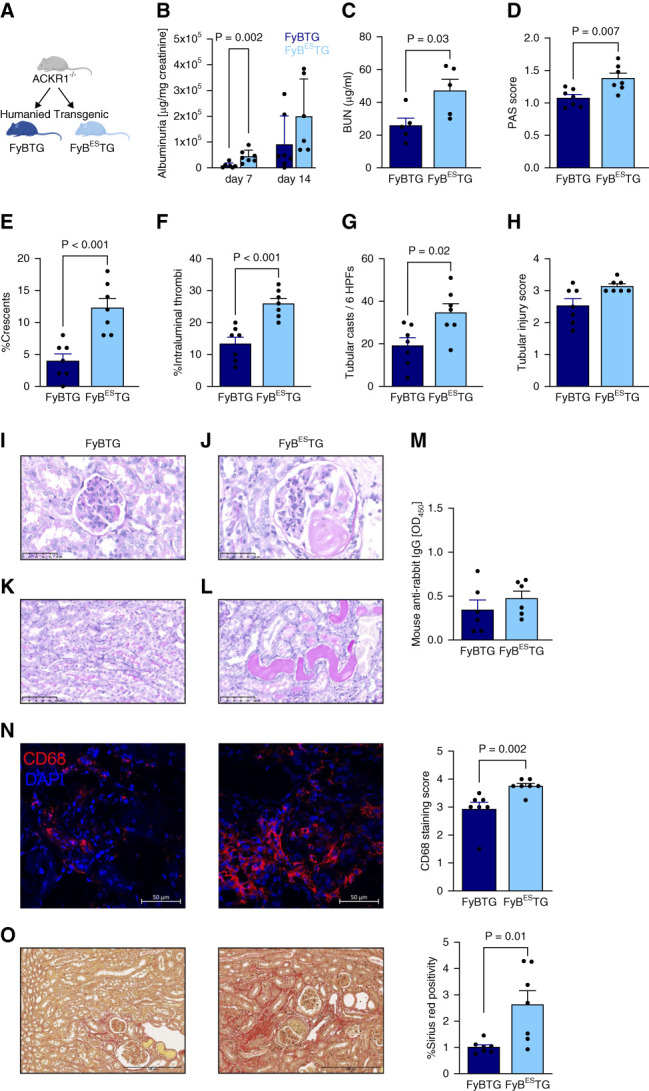
**Aggravated disease phenotype of nephrotoxic serum nephritis in humanized transgenic Duffy erythroid-silent mice with the erythroid-selective *ACKR1* deficiency.** (A) FyBTG (dark blue) and FyB^ES^TG mice (light blue) were subjected to 14 days of nephrotoxic serum nephritis. (B) Urinary albumin/creatinine ratio, (C) BUN, (D) glomerular PAS score, (E) glomerular crescent formation, (F) capillary intraluminal thrombi, (G) tubular casts, and (H) tubular injury score in FyBTG and FyB^ES^TG mice. Representative pictures of PAS-stained kidney sections from (I and K) FyBTG and (J and L) FyB^ES^TG mice. (M) 1:64,000 dilution of mouse anti-rabbit IgG in serum. (N) Kidneys were stained for CD68 (macrophages, red) and DAPI (nuclei, blue) and quantified. Representative sections are shown. (O) Representative micrographs and quantification of Picro-Sirius red–stained nephritic kidney sections from FyBTG and FyB^ES^TG mice. *n*=7 FyBTG (B, D–H, N, and O) and FyB^ES^TG (B day 7, D–H, N, and O); *n*=6 (FyB^ES^TG on day 14 in B and M) and *n*=5 (C) in each group from two independent experiments. (I, J, and N) Scale bars, 50 *µ*m. (K, L, and O) Scale bars, 100 *µ*m. All data are presented as mean+SEM. DAPI, 4′,6-diamidino-2-phenylindole; FyB, duffy B; FyB^ES^TG, FyB^ES^ transgene; FyBTG, FyB transgene.

We therefore analyzed the development of fibrotic changes in the kidneys of these mice by Picro-Sirius red staining, which revealed significantly increased fibrosis in the kidneys of erythroid-specific ACKR1-deficient mice (Figure 4M). Pathway enrichment analysis of differentially expressed genes in stromal cells of erythroid-specific ACKR1-deficient mice revealed a significant upregulation of pathways related to collagen formation and extracellular matrix organization. These transcriptomic changes support the observed increase in kidney fibrosis in these mice (Supplemental Figure 10).

### Generation and Characterization of a Transgenic Humanized Murine Model of Selective ACKR1 Deficiency on Erythrocytes

We next aimed to translate our findings in erythroid-specific ACKR1-deficient mice to the human situation. We therefore generated a mouse strain with human erythroid-selective ACKR1 deficiency and its ACKR1-sufficient controls. These strains carried transgenes for alternative human single-nucleotide *ACKR1* variants on the murine *Ackr1*^−/−^ background, encoding FyB (Caucasian) and FyB^(ES)^ (West African) Duffy blood groups, termed FyBTG and FyB^ES^TG, respectively (Figure 5A and Supplemental Figure 1). We confirmed relative human ACKR1 expression in different organs by quantitative PCR. Although no expression of human ACKR1 was detected in ACKR1-deficient mice, we found significant expression in the spleen, liver, lung, and heart and to a lesser extent also in the kidney, cerebellum, bone marrow, and skin in FyB^ES^TG mice (Supplemental Figure 11A). We additionally determined the presence of human ACKR1 on erythrocytes by flow cytometry using an anti-Fy6 antibody. The Fy6 signal was detected in erythrocytes of control FyBTG mice and human FyB-positive control erythrocytes, but not in ACKR1-deficient or FyB^ES^TG mice (Supplemental Figure 11B). Importantly, mouse ACKR1 was absent in erythrocytes of all genetically humanized mice (Supplemental Figure 11C). Human Fy6 antibody staining of spleen sections of FyBTG and FyB^ES^TG mice revealed venular EC staining in the red pulp, similar to that seen in human spleen (Supplemental Figure 11, D–G). Peripheral blood neutrophil counts in healthy mice were comparable between WT, ACKR1-deficient, and FyBTG mice but lower in FyB^ES^TG mice (Supplemental Figure 12).

### FyB^ES^TG Mice Developed Aggravated Inflammatory Kidney Disease

Finally, we induced nephrotoxic serum nephritis in FyBTG and FyB^ES^TG mice and examined disease parameters. Albuminuria, BUN, PAS score, crescent formation, capillary intraluminal thrombi, tubular casts, and tubular injury were all found to be significantly increased in FyB^ES^TG as compared with FyBTG mice (Figure 5, B–L). No changes were found in the humoral immune response measured by serum mouse anti-rabbit IgG (Figure 5M) and glomerular IgG deposition (Supplemental Table 1). As in erythroid ACKR1-deficient chimeric mice, infiltrating CD68^+^ macrophages (Figure 5N) and fibrosis (Figure 5O) were significantly increased in nephritic FyB^ES^TG kidneys.

## Discussion

In this study, we aimed at delineating the role of erythroid-expressed ACKR1 in the context of immune-mediated kidney disease. To this end, we employed different genetically modified mouse models to study the impact of the ACKR1 erythroid-deficient phenotype on the pathogenesis of nephrotoxic serum nephritis.

First, we evaluated global ACKR1-deficient mice and subjected them to the nephrotoxic serum nephritis model. Nephritis in these mice has previously been investigated by Vielhauer *et al.*,^38^ who noted accelerated development of the disease with increased humoral responses in the early phase and increased cell recruitment to the kidney, reaching significance in the later phases of the experimental disease. We also observed aggravated disease in ACKR1-deficient compared with WT mice, as evidenced by increased levels of albuminuria and exacerbated histopathologic indices, which is in agreement with previously reported findings in early disease; however, we did not detect changes in the humoral antibody response.^38^ Although we cannot fully account for this discrepancy, the observed temporal variations are most plausibly attributed to potential differences in the details of experimental setups.

To gain insights into the specific role of ACKR1 expressed on the erythroid lineage and to mirror the situation in Duffy-negative individuals of West African descent, we produced erythroid ACKR1-sufficient and ACKR1-deficient mice. Notably, we opted against the generation of endothelial ACKR1-deficient mice because of the absence of a corresponding human equivalent. ACKR1 expressed on nucleated erythroid cells regulates bone marrow myelopoiesis leading to neutropenia^33^ that develops because of the phenotypically altered neutrophils readily leaving circulation. Along with aggravated kidney disease and the major increase in kidney-infiltrating macrophages in erythroid-specific ACKR1-deficient mice, markers of activation and migration on classical and nonclassical monocytes showed a downregulation of CXCR4, CD16/32, and CD62L expression.^68,69^ Although CXCR4^hi^ monocytes are continuously retained in the bone marrow, CXCR4^lo^ monocytes are readily mobilized from the bone marrow into the blood.^70^ Of note, we also observed neutrophil shedding of CD62L, which is broadly perceived as an activation readout.^71^ The modified myeloid phenotypes have likely originated within the bone marrow, where ACKR1 is highly expressed on nucleated erythroid cells under WT conditions. Erythroid deficiency of ACKR1 in this niche is associated with alterations in the stem and progenitor cell compartment. *In vitro*, bone marrow–derived macrophages from ACKR1-deficient mice exhibited increased phagocytic capacity and were hyperreactive after stimulation with IL-4 or LPS. Thus, it is reasonable to speculate that ACKR1 expression on nucleated erythroid cells also influences monocytes in the bone marrow and that its absence leads to an activated phenotype that promotes migration. Because endothelial expression of ACKR1 in renal venular structures did not change in nephritic WT or erythroid-specific ACKR1-deficient mice, leukocytes can actively extravasate into the kidney tissue *via* ACKR1-dependent and ACKR1-independent mechanisms, which might contribute to the excessive macrophage abundance in kidney tissue. Proof-of-concept studies would be needed to clearly unravel the pathomechanism, which go far beyond the scope of this manuscript.

Depending on their marker characteristics, macrophages exert different (proinflammatory or anti-inflammatory) functions in inflammatory kidney disease, which also contribute to tissue fibrosis. Because the composition of macrophages infiltrating the kidneys of patients with lupus nephritis has been shown to be very heterogeneous,^72^ we aimed to evaluate the macrophage profiles in the kidney of nephritic mice expressing or lacking erythroid ACKR1 in an unbiased way using snRNAseq. Among others, we found *ApoE*, *Clusterin*, *Mertk*, and *Spp1* to be significantly upregulated in kidney-infiltrating myeloid cells of erythroid-specific ACKR1-deficient mice. These genes have previously been implicated in the differentiation of profibrotic macrophages.^64–67,73,74^

Although we cannot rule out the possibility that neutrophils contribute to the exacerbated kidney phenotype, we focused on macrophages because they were the most prominent infiltrating cell type in our study. We observed an increased amount of CD68^+^ApoE^+^, CD68^+^Clusterin^+^, and CD68^+^Spp1^+^ macrophages in nephritic erythroid-specific ACKR1-deficient kidneys. Consistent with the profibrotic macrophage phenotype, the kidneys of these mice displayed significantly higher levels of fibrosis. Fibroblasts were shown to be activated *via* SPP1 crosstalk,^75^ but the causality of macrophage changes that may contribute to the observed fibrotic phenotype in the kidney of these mice remains to be investigated.

To assess the significance and applicability of our findings in humans, we generated two transgenic humanized mouse strains (control FyB and Western Africa FyB^ES^) on the ACKR1-deficient background, which showed the same expression pattern as humans with the respective gene variants. The genetic predisposition of patients of West African ancestry to kidney diseases has attracted considerable attention in recent years. Patients with two APOL-1 variants and CKD face a higher risk of developing kidney failure because APOL-1 plays a pivotal role in podocyte homeostasis. However, these variants also protect against Trypanosoma infections.^76^ Similarly, ACKR1 deficiency on erythrocytes protects patients from malaria infections but aggravates immune-mediated kidney disease. Whether this mechanism also applies to other nonimmune kidney diseases or CKD in general needs to be investigated.

A major strength of this study lies in the use of multiple complementary mouse models of erythroid ACKR1 deficiency, including transgenic humanized mice, which consistently exhibited an aggravated phenotype in nephrotoxic serum nephritis. This multifaceted approach enhances the robustness and reproducibility of our findings. Notably, we provide the first experimental evidence linking the Duffy-negative phenotype to worsened kidney disease outcomes, thereby offering novel insights into the pathophysiologic consequences of ACKR1 deficiency in the context of immune-mediated kidney disease.

Our study has several limitations. First, we did not comprehensively elucidate the precise mechanisms of how ACKR1 deficiency impacts myeloid cell populations within the bone marrow niche. Second, we could not provide an unequivocal demonstration of macrophage contribution to disease exacerbation in the context of erythroid-specific ACKR1 deficiency. This can optimally be achieved by the depletion of macrophages in technically challenging experimentation, as would require *Ackr1*^−/−^ crosses with *LysM*-Cre/diphteria toxin receptor mice, the establishment of bone marrow chimeras, and their treatment with diphtheria toxin. Third, our findings warrant further validation in large human cohorts of patients with GN of West African ancestry, comparing those with Duffy-negative phenotype, FyB(ES), with Duffy-positive FyB patients. These studies ideally should incorporate both *in vitro* macrophage phenotyping and detailed assessment of pathologies in kidney biopsies.

Earlier reports have suggested a connection between ACKR1 and the development of experimental kidney disease.^37,38^ In summary, our data highlight the important role of ACKR1 expression on erythroid cells for immune homeostasis in the context of immune-mediated kidney disease. Selective deficiency of ACKR1 on erythroid cells was associated with aggravated nephrotoxic serum nephritis as shown in two different genetically modified mouse models. We conclude that functional myeloid cell changes downstream of erythroid ACKR1 deficiency cause a profibrotic and activated phenotype of infiltrating macrophages and, consequently, increased fibrosis and kidney damage. By using new transgenic humanized mouse strains that express human FyB or FyB^ES^ on an *Ackr1*^−/−^ background, we proved that our findings are reproducible when human transcripts of two alternative *ACKR1* single-nucleotide polymorphisms are present. We were able to demonstrate the pathomechanistic contribution of selective ACKR1 deficiency on erythroid cells, which is of paramount clinical relevance for patients of West African descent suffering from immune-mediated kidney disease.

## Supplementary Material

## Data Availability

Original data generated for the study are available in a public access repository. Data Type: Raw Data/Source Data. Repository Name: Gene Expression Omnibus. Linkable Citation: https://www.ncbi.nlm.nih.gov/geo/query/acc.cgi?acc=GSE276017. Data from the snRNAseq are deposited in Gene Expression Omnibus with the accession code GSE276017.

## Declarative Statements

All animal experiments were conducted in accordance with the NIH Guide for the Care and Use of Laboratory Animals or an equivalent standard that meets or exceeds the ethical and welfare requirements outlined in the NIH Guide. All protocols were approved by the appropriate institutional animal care and use committee.

## Supplemental Material

Supplemental Methods

Supplemental Figure 1. Map of cloned construct for the generation of transgenic humanized mice.

Supplemental Figure 2. Disease phenotype in nephritic WT and Ackr1^−/−^ mice.

Supplemental Figure 3. Successful bone marrow chimerism.

Supplemental Figure 4. Erythroid ACKR1 deletion does not affect T-cell immunity and regulatory differentiation.

Supplemental Figure 5. Gating strategy for classical and nonclassical monocytes as well as neutrophils.

Supplemental Figure 6. ACKR1-deficient bone marrow–derived macrophages are hyperreactive.

Supplemental Figure 7. ACKR1 is not expressed in murine glomeruli.

Supplemental Figure 8. Immune subclustering marker genes.

Supplemental Figure 9. Relative proportions of major cell populations.

Supplemental Figure 10. Functional pathway analysis in stromal and proximal tubular cells.

Supplemental Figure 11. Erythrocyte ACKR1 expression in ACKR1^−/−^, FyB^ES^TG and respective controls.

Supplemental Figure 12. Differential blood leukocyte and blood neutrophil count.

Supplemental Table 1. Autologous antibody deposition in glomeruli of nephritic mice.

Supplemental Table 2. Gene expression of myeloid cells, stromal cells, and proximal tubular cells in chimeric WT mice reconstituted with WT cells and ACKR1^−/−^ cells.

## References

[1] Price-Haywood EG Burton J Fort D Seoane L. Hospitalization and mortality among Black patients and white patients with Covid-19. N Engl J Med. 2020;382(26):2534–2543. doi:10.1056/nejmsa2011686 32459916 PMC7269015

[2] Rentsch CT Kidwai-Khan F Tate JP, . Patterns of COVID-19 testing and mortality by race and ethnicity among United States veterans: a nationwide cohort study. PLoS Med. 2020;17(9):e1003379. doi:10.1371/journal.pmed.1003379 32960880 PMC7508372

[3] Dombrovskiy VY Martin AA Sunderram J Paz HL. Occurrence and outcomes of sepsis: influence of race. Crit Care Med. 2007;35(3):763–768. doi:10.1097/01.ccm.0000256726.80998.bf 17255870

[4] Samus M Rot A. Atypical chemokine receptors in cancer. Cytokine. 2024;176:156504. doi:10.1016/j.cyto.2024.156504 38266462

[5] US Renal Data System 2019 annual data report: epidemiology of kidney disease in the United States. Am J Kidney Dis. 2019;75(1):S1–S64. doi:10.1053/j.ajkd.2019.09.002 31704083

[6] Kaze AD Ilori T Jaar BG Echouffo-Tcheugui JB. Burden of chronic kidney disease on the African continent: a systematic review and meta-analysis. BMC Nephrol. 2018;19(1):125. doi:10.1186/s12882-018-0930-5 29859046 PMC5984759

[7] Hung RKY Santana-Suarez B Binns-Roemer E, . The epidemiology of kidney disease in people of African ancestry with HIV in the UK. EClinicalMedicine. 2021;38:101006. doi:10.1016/j.eclinm.2021.101006 34286237 PMC8273351

[8] George C Stoker S Okpechi I Woodward M Kengne A.; CKD-Africa Collaboration. The chronic kidney disease in Africa (CKD-Africa) collaboration: lessons from a new pan-African network. BMJ Glob Health. 2021;6(8):e006454. doi:10.1136/bmjgh-2021-006454 PMC8340290 34348933

[9] Lanata CM Nititham J Taylor KE, . Genetic contributions to lupus nephritis in a multi-ethnic cohort of systemic lupus erythematous patients. PLoS One. 2018;13(6):e0199003. doi:10.1371/journal.pone.0199003 29953444 PMC6023154

[10] Svetkey LP McKeown SP Wilson AF. Heritability of salt sensitivity in Black Americans. Hypertension. 1996;28(5):854–858. doi:10.1161/01.hyp.28.5.854 8901834

[11] Lackland DT. Racial differences in hypertension: implications for high blood pressure management. Am J Med Sci. 2014;348(2):135–138. doi:10.1097/maj.0000000000000308 24983758 PMC4108512

[12] Klimentidis YC Arora A Zhou J Kittles R Allison DB. The genetic contribution of West-African ancestry to protection against central obesity in African-American men but not women: results from the ARIC and MESA studies. Front Genet. 2016;7:89. doi:10.3389/fgene.2016.00089 27313598 PMC4888933

[13] Tekola-Ayele F Adeyemo AA Rotimi CN. Genetic epidemiology of type 2 diabetes and cardiovascular diseases in Africa. Prog Cardiovasc Dis. 2013;56(3):251–260. doi:10.1016/j.pcad.2013.09.013 24267432 PMC3840391

[14] Kopp JB Winkler CA Zhao X, . Clinical features and histology of apolipoprotein L1-Associated nephropathy in the FSGS clinical trial. J Am Soc Nephrol. 2015;26(6):1443–1448. doi:10.1681/ASN.2013111242 25573908 PMC4446865

[15] Swanepoel CR Atta MG D’Agati VD, . Kidney disease in the setting of HIV infection: conclusions from a Kidney Disease: Improving Global Outcomes (KDIGO) Controversies Conference. Kidney Int. 2018;93(3):545–559. doi:10.1016/j.kint.2017.11.007 29398134 PMC5983378

[16] Cutbush M Mollison PL. The Duffy blood group system. Heredity. 1950;4(3):383–389. doi:10.1038/hdy.1950.31 14802995

[17] Thiriot A Perdomo C Cheng G, . Differential DARC/ACKR1 expression distinguishes venular from non-venular endothelial cells in murine tissues. BMC Biol. 2017;15(1):45. doi:10.1186/s12915-017-0381-7 28526034 PMC5438556

[18] Peiper SC Wang ZX Neote K, . The Duffy antigen/receptor for chemokines (DARC) is expressed in endothelial cells of Duffy negative individuals who lack the erythrocyte receptor. J Exp Med. 1995;181(4):1311–1317. doi:10.1084/jem.181.4.1311 7699323 PMC2191961

[19] Horuk R Martin A Hesselgesser J, . The Duffy antigen receptor for chemokines: structural analysis and expression in the brain. J Leukoc Biol. 1996;59(1):29–38. doi:10.1002/jlb.59.1.29 8558064

[20] Rot A Gutjahr JC Biswas A, . Murine bone marrow macrophages and human monocytes do not express atypical chemokine receptor 1. Cell Stem Cell. 2022;29(7):1013–1015. doi:10.1016/j.stem.2021.11.010 35803222

[21] Howes RE Patil AP Piel FB, . The global distribution of the Duffy blood group. Nat Commun. 2011;2(1):266. doi:10.1038/ncomms1265 21468018 PMC3074097

[22] Tournamille C Colin Y Cartron JP Le Van Kim C. Disruption of a GATA motif in the Duffy gene promoter abolishes erythroid gene expression in Duffy–negative individuals. Nat Genet. 1995;10(2):224–228. doi:10.1038/ng0695-224 7663520

[23] Bruggeman LA O’Toole JF Sedor JR. APOL1 polymorphisms and kidney disease: loss-of-function or gain-of-function? Am J Physiol Renal Physiol. 2019;316(1):F1–F8. doi:10.1152/ajprenal.00426.2018 30332315 PMC6383195

[24] Miller LH Mason SJ Clyde DF McGinniss MH. The resistance factor to plasmodium vivax in blacks — the duffy-blood-group genotype, FyFy. N Engl J Med. 1976;295(6):302–304. doi:10.1056/nejm197608052950602 778616

[25] Szabo MC Soo KS Zlotnik A Schall TJ. Chemokine class differences in binding to the Duffy antigen-erythrocyte chemokine receptor. J Biol Chem. 1995;270(43):25348–25351. doi:10.1074/jbc.270.43.25348 7592697

[26] Gutjahr JC Crawford KS Jensen DR, . The dimeric form of CXCL12 binds to atypical chemokine receptor 1. Sci Signal. 2021;14(696):eabc9012. doi:10.1126/scisignal.abc9012 34404752 PMC9015690

[27] Fukuma N Akimitsu N Hamamoto H Kusuhara H Sugiyama Y Sekimizu K. A role of the Duffy antigen for the maintenance of plasma chemokine concentrations. Biochem Biophys Res Commun. 2003;303(1):137–139. doi:10.1016/s0006-291x(03)00293-6 12646177

[28] Pruenster M Mudde L Bombosi P, . The Duffy antigen receptor for chemokines transports chemokines and supports their promigratory activity. Nat Immunol. 2009;10(1):101–108. doi:10.1038/ni.1675 19060902 PMC3205989

[29] Middleton J Neil S Wintle J, . Transcytosis and surface presentation of IL-8 by venular endothelial cells. Cell. 1997;91(3):385–395. doi:10.1016/s0092-8674(00)80422-5 9363947

[30] Novitzky-Basso I Rot A. Duffy antigen receptor for chemokines and its involvement in patterning and control of inflammatory chemokines. Front Immunol. 2012;3:266. doi:10.3389/fimmu.2012.00266 22912641 PMC3421148

[31] Jilma-Stohlawetz P Homoncik M Drucker C, . Fy phenotype and gender determine plasma levels of monocyte chemotactic protein. Transfusion. 2001;41(3):378–381. doi:10.1046/j.1537-2995.2001.41030378.x 11274594

[32] Darbonne WC Rice GC Mohler MA, . Red blood cells are a sink for interleukin 8, a leukocyte chemotaxin. J Clin Invest. 1991;88(4):1362–1369. doi:10.1172/jci115442 1918386 PMC295607

[33] Duchene J Novitzky-Basso I Thiriot A, . Atypical chemokine receptor 1 on nucleated erythroid cells regulates hematopoiesis. Nat Immunol. 2017;18(7):753–761. doi:10.1038/ni.3763 28553950 PMC5480598

[34] Segerer S Regele H Mack M, . The Duffy antigen receptor for chemokines is up-regulated during acute renal transplant rejection and crescentic glomerulonephritis. Kidney Int. 2000;58(4):1546–1556. doi:10.1046/j.1523-1755.2000.00316.x 11012889

[35] Segerer S Böhmig GA Exner M, . When renal allografts turn DARC. Transplantation. 2003;75(7):1030–1034. doi:10.1097/01.tp.0000054679.91112.6f 12698093

[36] Akalin E Neylan JF. The influence of Duffy blood group on renal allograft outcome in African Americans. Transplantation. 2003;75(9):1496–1500. doi:10.1097/01.tp.0000061228.38243.26 12792503

[37] Zarbock A Schmolke M Bockhorn SG, . The Duffy antigen receptor for chemokines in acute renal failure: a facilitator of renal chemokine presentation. Crit Care Med. 2007;35(9):2156–2163. doi:10.1097/01.ccm.0000280570.82885.32 17855830

[38] Vielhauer V Allam R Lindenmeyer MT, . Efficient renal recruitment of macrophages and T cells in mice lacking the duffy antigen/receptor for chemokines. Am J Pathol. 2009;175(1):119–131. doi:10.2353/ajpath.2009.080590 19498001 PMC2708800

[39] Eller K Weber T Pruenster M, . CCR7 deficiency exacerbates injury in acute nephritis due to aberrant localization of regulatory T cells. J Am Soc Nephrol. 2010;21(1):42–52. doi:10.1681/ASN.2009020133 19917782 PMC2799279

[40] Wolf D Hochegger K Wolf AM, . CD4+CD25+ regulatory T cells inhibit experimental anti-glomerular basement membrane glomerulonephritis in mice. J Am Soc Nephrol. 2005;16(5):1360–1370. doi:10.1681/ASN.2004100837 15788479

[41] Kurts C Panzer U Anders HJ Rees AJ. The immune system and kidney disease: basic concepts and clinical implications. Nat Rev Immunol. 2013;13(10):738–753. doi:10.1038/nri3523 24037418

[42] Artinger K Kirsch AH Aringer I, . Innate and adaptive immunity in experimental glomerulonephritis: a pathfinder tale. Pediatr Nephrol. 2017;32(6):943–947. doi:10.1007/s00467-016-3404-7 27169420 PMC5399043

[43] Dawson TC Lentsch AB Wang Z, . Exaggerated response to endotoxin in mice lacking the Duffy antigen/receptor for chemokines (DARC). Blood. 2000;96(5):1681–1684. doi:10.1182/blood.v96.5.1681 10961863

[44] Chaudhuri A Yuen G Fang F Storry J. Development of Duffy transgenic mouse: in vivo expression of human Duffy gene with −33T→C promoter mutation in non‐erythroid tissues. Br J Haematol. 2004;127(3):356–359. doi:10.1111/j.1365-2141.2004.05208.x 15491299

[45] Rosenkranz AR Mendrick DL Cotran RS Mayadas TN. P-selectin deficiency exacerbates experimental glomerulonephritis: a protective role for endothelial P-selectin in inflammation. J Clin Invest. 1999;103(5):649–659. doi:10.1172/jci5183 10074481 PMC408121

[46] Janssen U Ostendorf T Gaertner S, . Improved survival and amelioration of nephrotoxic nephritis in intercellular adhesion molecule-1 knockout mice. J Am Soc Nephrol. 1998;9(10):1805–1814. doi:10.1681/ASN.v9101805 9773781

[47] Schrijver G Bogman MJ Assmann KJ, . Anti-GBM nephritis in the mouse: role of granulocytes in the heterologous phase. Kidney Int. 1990;38(1):86–95. doi:10.1038/ki.1990.171 2385089

[48] Zheng GXY Terry JM Belgrader P, . Massively parallel digital transcriptional profiling of single cells. Nat Commun. 2017;8(1):14049. doi:10.1038/ncomms14049 28091601 PMC5241818

[49] Lopez R Regier J Cole MB Jordan MI Yosef N. Deep generative modeling for single-cell transcriptomics. Nat Methods. 2018;15(12):1053–1058. doi:10.1038/s41592-018-0229-2 30504886 PMC6289068

[50] Wolf FA Angerer P Theis FJ. SCANPY: large-scale single-cell gene expression data analysis. Genome Biol. 2018;19(1):15. doi:10.1186/s13059-017-1382-0 29409532 PMC5802054

[51] Stuart T Butler A Hoffman P, . Comprehensive integration of single-cell data. Cell. 2019;177(7):1888–1902.e21. doi:10.1016/j.cell.2019.05.031 31178118 PMC6687398

[52] Paust HJ Turner JE Steinmetz OM, . The IL-23/Th17 axis contributes to renal injury in experimental glomerulonephritis. J Am Soc Nephrol. 2009;20(5):969–979. doi:10.1681/ASN.2008050556 19339380 PMC2678032

[53] Faul F Erdfelder E Lang AG Buchner A. G*Power 3: a flexible statistical power analysis program for the social, behavioral, and biomedical sciences. Behav Res Methods. 2007;39(2):175–191. doi:10.3758/bf03193146 17695343

[54] Ferreira FM Palle P Vom Berg J Prajwal P Laman JD Buch T. Bone marrow chimeras—a vital tool in basic and translational research. J Mol Med. 2019;97(7):889–896. doi:10.1007/s00109-019-01783-z 31028417

[55] Balzer MS Rohacs T Susztak K. How many cell types are in the kidney and what do they do? Annu Rev Physiol. 2022;84(1):507–531. doi:10.1146/annurev-physiol-052521-121841 34843404 PMC9233501

[56] Lake BB Menon R Winfree S, . An atlas of healthy and injured cell states and niches in the human kidney. Nature. 2023;619(7970):585–594. doi:10.1038/s41586-023-05769-3 37468583 PMC10356613

[57] Uhlén M Fagerberg L Hallström BM, . Proteomics. Tissue-based map of the human proteome. Science. 2015;347(6220):1260419. doi:10.1126/science.1260419 25613900

[58] Caberoy NB Alvarado G Li W. Tubby regulates microglial phagocytosis through MerTK. J Neuroimmunol. 2012;252(1-2):40–48. doi:10.1016/j.jneuroim.2012.07.009 22884297 PMC3466361

[59] Paone C Rodrigues N Ittner E Santos C Buntru A Hauck CR. The tyrosine kinase Pyk2 contributes to complement-mediated phagocytosis in murine macrophages. J Innate Immun. 2016;8(5):437–451. doi:10.1159/000442944 26848986 PMC6738876

[60] Lin C Rezaee F Waasdorp M, . Protease activated receptor-1 regulates macrophage-mediated cellular senescence: a risk for idiopathic pulmonary fibrosis. Oncotarget. 2015;6(34):35304–35314. doi:10.18632/oncotarget.6095 26474459 PMC4742106

[61] Jin J Wang Y Ma Q, . LAIR-1 activation inhibits inflammatory macrophage phenotype in vitro. Cell Immunol. 2018;331:78–84. doi:10.1016/j.cellimm.2018.05.011 29887420

[62] Kittikulsuth W Nakano D Kitada K, . Vasoactive intestinal peptide blockade suppresses tumor growth by regulating macrophage polarization and function in CT26 tumor-bearing mice. Sci Rep. 2023;13(1):927. doi:10.1038/s41598-023-28073-6 36650220 PMC9845384

[63] Xu Y Hillman H Chang M, . Identification of conserved and tissue-restricted transcriptional profiles for lipid associated macrophages. Commun Biol. 2025;8(1):953. doi:10.1038/s42003-025-08387-z 40550904 PMC12185701

[64] Morse C Tabib T Sembrat J, . Proliferating SPP1/MERTK-expressing macrophages in idiopathic pulmonary fibrosis. Eur Respir J. 2019;54(2):1802441. doi:10.1183/13993003.02441-2018 31221805 PMC8025672

[65] Tan Y Zhao L Yang YG Liu W. The role of osteopontin in tumor progression through tumor-associated macrophages. Front Oncol. 2022;12:953283. doi:10.3389/fonc.2022.953283 35898884 PMC9309262

[66] Baitsch D Bock HH Engel T, . Apolipoprotein E induces antiinflammatory phenotype in macrophages. Arterioscler Thromb Vasc Biol. 2011;31(5):1160–1168. doi:10.1161/atvbaha.111.222745 21350196 PMC3529398

[67] Weng X Zhao H Guan Q, . Clusterin regulates macrophage expansion, polarization and phagocytic activity in response to inflammation in the kidneys. Immunol Cell Biol. 2021;99(3):274–287. doi:10.1111/imcb.12405 32935392 PMC7984284

[68] Rzeniewicz K Newe A Rey Gallardo A, . L-selectin shedding is activated specifically within transmigrating pseudopods of monocytes to regulate cell polarity in vitro. Proc Natl Acad Sci U S A. 2015;112(12):E1461–E1470. doi:10.1073/pnas.1417100112 25775539 PMC4378423

[69] Rey-Gallardo A Tomlins H Joachim J, . Sequential binding of ezrin and moesin to L-selectin regulates monocyte protrusive behaviour during transendothelial migration. J Cell Sci. 2018;131(13):jcs215541. doi:10.1242/jcs.215541 29777033 PMC6051341

[70] Chong SZ Evrard M Devi S, . CXCR4 identifies transitional bone marrow premonocytes that replenish the mature monocyte pool for peripheral responses. J Exp Med. 2016;213(11):2293–2314. doi:10.1084/jem.20160800 27811056 PMC5068243

[71] Ivetic A Hoskins Green HL Hart SJ. L-selectin: a major regulator of leukocyte adhesion, migration and signaling. Front Immunol. 2019;10:1068. doi:10.3389/fimmu.2019.01068 31139190 PMC6527602

[72] Kwant LE Vegting Y Tsang-a-Sjoe MWP, . Macrophages in lupus nephritis: exploring a potential new therapeutic avenue. Autoimmun Rev. 2022;21(12):103211. doi:10.1016/j.autrev.2022.103211 36252930

[73] Pastore M Caligiuri A Raggi C, . Macrophage MerTK promotes profibrogenic cross-talk with hepatic stellate cells via soluble mediators. JHEP Rep. 2022;4(4):100444. doi:10.1016/j.jhepr.2022.100444 35252828 PMC8891698

[74] She Y Xu X Yu Q Yang X He J Tang XX. Elevated expression of macrophage MERTK exhibits profibrotic effects and results in defective regulation of efferocytosis function in pulmonary fibrosis. Respir Res. 2023;24(1):118. doi:10.1186/s12931-023-02424-3 37120511 PMC10148433

[75] Hoeft K Schaefer GJL Kim H, . Platelet-instructed SPP1+ macrophages drive myofibroblast activation in fibrosis in a CXCL4-dependent manner. Cell Rep. 2023;42(2):112131. doi:10.1016/j.celrep.2023.112131 36807143 PMC9992450

[76] Egbuna O Zimmerman B Manos G, . Inaxaplin for proteinuric kidney disease in persons with two APOL1 variants. N Engl J Med. 2023;388(11):969–979. doi:10.1056/nejmoa2202396 36920755

