# Supplementary material for: Erythroid Atypical Chemokine Receptor 1 Deficiency Aggravates Immune-Mediated Kidney Disease
Source: J Am Soc Nephrol. 2025 Sep 17;37(4):731–46. doi: 10.1681/ASN.0000000878 (PMC13065172; doi:10.1681/ASN.0000000878)
Supplement: Supplementary file 2 [file jasn-37-731-s002.pdf]

Supplemental Material

Supplemental Figure 1 – Map of cloned construct for the generation of transgenic humanized mice
Supplemental Figure 2– Disease phenotype in nephritic WT and Ackr1-/- mice
Supplemental Figure 3 - Successful bone marrow chimerism
Supplemental Figure 4– Erythroid ACKR1 deletion does not impact T cell immunity and regulatory differentiation
Supplemental Figure 5 – Gating strategy for classical and non-classical monocytes as well as neutrophils
Supplemental Figure 6– ACKR1-deficient bone marrow-derived macrophages are hyperreactive
Supplemental Figure 7 - ACKR1 is not expressed in murine glomeruli
Supplemental Figure 8 – Immune subclustering marker genes
Supplemental Figure 9 – Relative proportions of major cell populations
Supplemental Figure 10 – Functional pathway analysis in stromal and proximal tubular cells
Supplemental Figure 11 – Erythrocyte ACKR1 expression in ACKR1-/-, FYBESTG and respective controls
Supplemental Figure 12 – Differential blood leukocyte and blood neutrophil count
Supplemental Table 1 – Autologous antibody deposition in glomeruli of nephritic mice
Supplemental Table 2 – Gene expression of myeloid cells, stromal cells and proximal tubular cells in chimeric WT mice reconstituted with WT cells and ACKR1-/- cells

SUPPLEMENTAL METHODS

Generation of bone marrow chimeric mice

To produce selective erythroid ACKR1-deficient and their control erythroid ACKR1-sufficient mice, bone marrow from either 8-week-old male C57BL/6J wild type (WT) or ACKR1-deficient mice on the C57BL/6J background was adoptively transferred into 8-week-old male WT recipients within 24 hours of their irradiation with 8 Gy.[1] Each mouse received $5x10^6$ bone marrow cells in 100 µl PBS injected via the tail vein followed by prophylactic enrofloxacin (Baytril, Bayer AG, Germany) starting on the day of irradiation. Mice were allowed to recover for 8 weeks, chimerism was confirmed by PCR and flow cytometry (Supplemental Figure 3), and NTSN was induced as described in the main methods.

Establishment of a transgenic, humanized FyBTG and $FyB^{ES}TG$ mouse model

The transgenesis was performed as described previously[2], with a key difference of micro-injecting human transgenes into fertilized oocytes isolated from super-ovulated *Ackr1*$^{-/-}$ instead of WT mice. Importantly, the entire *ACKR1* gene with all potential regulatory regions was included, specifically incorporating 1032 bp before and 1241 bp after the open reading frame of the main exon (Supplemental Figure 1). For this, human DNA was extracted from suitable homozygous donors using the DNeasy Blood & Tissue Kit (Qiagen, Hilden, Germany). Cloning of human DNA was undertaken using the High-Fidelity Phusion DNA polymerase (Thermo Fisher Scientific, Waltham, MA, USA) according to the manufacturer´s protocol. Forward primer: ACA TGG TTT GAA CTG CCT TTC C; Reverse primer: CCT CTG GGT AGA GGG TGA ATT TGC, giving a product of 3203 bp that was cloned into the pJET1.2-vector (Fermentas, Burlington, Ontario, Canada) and used for transformation of Gold Efficiency α-Select chemically competent cells (Bioline, Memphis, TN, USA).

Plasmid DNA was extracted using the Qiagen Plasmid Maxi kit. The amount of DNA in the preparation was routinely determined by measuring OD_{260} on a spectrophotometer for amplification. The vector backbone was removed using 20 µl of 5x Tango buffer, Xba1 30 units (New England Biosciences, Ipswich, MA, USA), Xho1 15 units (New England Biosciences), PVU1 15 units (Promega, Madison, WI, USA), and 6 5µl ddH_2O overnight at 37°C (16 hours). Subsequently, DNA from one digest was purified using the Fermentas Purification Kit (Fermentas). The digested product was run on a 0.7% agarose gel and revealed the presence of predicted bands. The product was purified through Fermentas GeneJET PCR Purification Kit (Fermentas) according to the manufacturer´s instructions and analyzed by EcoRI restriction enzyme digest. Sequencing was performed at the University of Birmingham School of Biosciences Sequencing Service and sequence data were extracted using Sequence Scanner 1.0 (Applied Biosystems, Waltham, MA, USA). Initial screening confirmed the presence of T-46C GATA-1 promoter mutation and the presence of A at position 1142, indicating that the FyB^{ES} ACKR1 allele was incorporated in the FyB^{ES} clone. For FyBTG mice, the absence of T-46C in the GATA promoter, and the presence of A1142 were confirmed, indicating that the FyB ACKR1 allele was incorporated in clones.

Clodronate treatment

ACKR1-deficient mice were administered 50mg/kg bodyweight of control liposomes or clodronate liposomes via intraperitoneal injections every 75 hours beginning on the day of NTSN induction.

Evaluation of Urea Nitrogen

A commercially available colorimetric detection kit was used according to the manufacturer´s instructions (Invitrogen, Waltham, MA, USA). Absorbance was read at 450 nm.

snRNAseq analysis

Preprocessing and data cleaning: Cell Ranger output files were further processed using SEURAT.[3] Samples were pre-processed and cleaned of unwanted cellular events to match a number of genes between 200 – 4000 and a percentage of mitochondrial genes <5%. Data sets were merged with the SEURAT v3 VST-integration workflow for initial clustering (Louvain). Low-quality clusters were removed based on enrichment for mitochondrial and ribosomal genes or generally low count/feature numbers, while missing expression of informative marker genes. Cells from one control sample were excluded due to quality concerns. *Clustering and annotation*: After initial processing and quality control, the dataset was converted to an AnnData object and further analyzed using Scanpy (v1.9.5).[4] For cell type annotation, cells were clustered using the sc.tl.leiden() function, followed by sc.tl.rank_genes_groups() to identify marker genes for each cluster. Leiden clusters were grouped and annotated based on informative marker genes. Final cell type annotations were projected onto the UMAP embedding. *Differential expression analysis*: Differential expression analysis was performed using the non-parametric Wilcoxon test implemented in sc.tl.rank_genes_groups(). Differentially expressed genes (DEGs) were computed separately for each cell type. *Iterative immune cell subclustering*: For subclustering of immune cells, the analysis pipeline was adapted and repeated using immune cells only. The final embedding was generated based on 3000 highly variable genes identified via sc.pp.highly_variable_genes() and integrated using scVI (v1.0.3).[5] Neighborhood graph construction was based on n_pcs =

100 and visualized using UMAP. Functional pathway analysis was performed using the pathway enrichment tool ShinyGO 0.82[6], considering the Gene Ontology molecular function[7,8] , Biocarta[9] and Reactome[10] databases. All pathway analyses are based on upregulated genes and only significant pathways (FDR <0.1) are shown.

Immunofluorescence

After blocking with 20% goat serum, the slides were incubated at room temperature with directly conjugated antibodies against ACKR1,[11] PECAM-1 (clone 390 and MEC13.1; Biolegend, San Diego, CA, USA), Podoplanin (clone 8.1.1, Biolegend), ApoE (clone 1B2C9, Proteintech, Rosemont, IL, USA), F4/80 (clone BM8, Biolegend), FITC-conjugated goat anti-mouse IgG (Jackson ImmunoResearch Laboratories, West Grove, PA, USA), or unconjugated primary antibodies against CD4 (clone YTS191.1; Serotec, Oxford, UK), CD8 (Clone KT15; Serotec), CD68 (clone FA-11; Serotec), Ly6G antibody (clone 1A8, Abcam, Cambridge, MA, USA), Osteopontin (ab11503, Abcam), Apolipoprotein J (PA5-46931, Invitrogen), anti-liver Arginase (ab91279, Abcam), or anti-human Fy6 (mouse IgG2b, a kind gift from Dr. M Uchikawa, Japanese Red Cross) diluted in 1xPBS/0.1%BSA solution in a moist chamber for 30-60 minutes at room temperature or overnight at 4°C in the dark. After washing in PBS, the slides stained with unconjugated primary antibodies were subsequently stained with the secondary fluorescently labeled antibodies donkey anti-rat Alexa Fluor 488 (AB_2535794, Invitrogen), donkey anti-rat Alexa Fluor 555 (AB_2910652, Invitrogen), and donkey anti-goat Alexa Fluor 555 (AB_2535853, Invitrogen) for 45 minutes, washed again, and counterstained with DAPI for five minutes.

Evaluation of histopathology

For evaluation of periodic acid-Schiff (PAS) stained kidney sections,[12] a minimum of 50 glomerular cross-sections were analyzed. Shortly, PAS^+ material was scored within glomeruli following a semiquantitative scoring system consecutively numbered 0-3. Crescents (defined as > 2 cell layers) as well as intraluminal capillary thrombi were classified in a minimum of 50 glomeruli. Tubular casts were quantified per 6 high power fields (40x) and the acute tubular injury score[13] was determined by the percentage of tubular dilation, loss of brush border, cell necrosis, and cast formation in at least 10 high power fields as follows: 0= no tubular injury, 1≤10%; 2: 11-25%; 3: 26-45%; 4: 46-75%; 5>76%. The percentages of Picro-Sirius red positivity were evaluated using a macro for ImageJ. When evaluating kidney infiltrating cells, cell numbers of kidney infiltrating $CD4^+$ T cells, $CD8^+$ T cells, $F4/80^+$ cells and $Ly6G^+$ neutrophils were counted in six high-power fields of renal cortex and medulla, while infiltrating macrophages were evaluated using a semiquantitative scoring system as listed below: 0 = 0 to 4 cells stained positive, 1+ = 5 to 10 cells, 2+ = 11 to 50 cells, 3+ = 51 to 200 cells, and 4+ >200 cells stained positive per low-power field (20x). Percentages of ApoE, Clusterin and Spp1-expressing macrophages were determined using QuPath.

Autologous antibody response

Plates were incubated overnight with 100µg/ml rabbit IgG (Jackson ImmunoResearch Laboratories). Serum was then incubated with serial-doubling dilutions. HRP-conjugated anti-mouse IgG was used for the detection of circulating mouse anti-rabbit immunoglobulin.

Reverse transcription real-time polymerase chain reaction

Total RNA was extracted using TRI Reagent (Sigma-Aldrich) or RNeasy kit (Qiagen). Subsequently, cDNA was synthesized by using Superscript III Transcription Kit (Invitrogen) and random primers (Invitrogen) or SuperScript VILO cDNA Synthesis Kit (Invitrogen) for reverse transcription of 1 µg or 2 µg of total RNA. Real-time polymerase chain reaction was performed in duplicate on a CFX96 Real-Time System (BioRad, Hercules, CA, USA). TaqMan gene expression assays (Applied Biosystems) Mm00444540_m1 for *CD86*, Mm00450960_m1 for *Tbx21*, Mm00801778_m1 for *Ifn-y*, and Mm01261022_m1 for *Rorc* were used. *HPRT* served as a housekeeping gene. The threshold cycle values were then used to calculate fold change relative to the housekeeping gene using the $2^{-\Delta\Delta CT}$ method or the reciprocal of the difference in Ct value between the target and the housekeeping gene.

Flow cytometry staining

The erythrocytes were stained with fluorescent antibodies by diluting whole blood so that $1x10^6$ erythrocytes were present in 100 µl of wash buffer. The samples were incubated with heat-inactivated FCS and purified anti-mouse or -human CD16/CD32, centrifuged at 375xg for 10 min and then incubated with an appropriate antibody mix (anti-ACKR1 conjugated to Alexa-Fluor 488 or Alexa-Fluor 647). Alternatively, erythrocytes were incubated with anti-Fy6 for 30 min at 4-8°C, washed, and incubated with goat anti-mouse IgG-PE for 20 min at 4-8°C. For the determination of absolute and differential leukocyte counts, red blood cells were lysed using 1x BD Pharmlyse (BD Bioscience, Franklin Lakes, NJ, USA). The full blood count was obtained by the automated ABX Pentra 60 (HORIBA ABX S.A.S., Northampton, UK) blood counter.

For the staining of leukocyte populations from lymphoid tissue, bone marrow, and blood, a single-cell suspension was obtained using 70-µm cell strainers. Cell isolation from kidneys was

performed using Percoll density gradient centrifugation after digestion of finely minced kidneys in RPMI 1640 medium (Thermo Fisher Scientific) supplemented with collagenase D and DNAse I at 37°C. For intracellular staining, the True-Nuclear Transcription Factor Buffer Set (Biolegend) was used. Cells were then stained with antibodies against ACKR1,[11] Ly6G, CD11b, CD3 (BD Bioscience), CD45, CD8, CD25, Foxp3, CD4, CD11b, Ly6G, Ly6C, CD16/32, CD62L, CXCR4 (Biolegend), F4/80, Ter-119, CD115 and with Fixable Viability Dye (Invitrogen) and washed before analysis.

SUPPLEMENTARY REFERENCES

1. Duchene J, Novitzky-Basso I, Thiriot A, et al. Atypical chemokine receptor 1 on nucleated erythroid cells regulates hematopoiesis. *Nat Immunol*. 2017;18(7):753-761. doi:10.1038/ni.3763

2. Chaudhuri A, Yuen G, Fang F, Storry J. Development of Duffy transgenic mouse: in vivo expression of human Duffy gene with −33T→C promoter mutation in non-erythroid tissues. *Br J Haematol*. 2004;127(3):356-359. doi:10.1111/j.1365-2141.2004.05208.x

3. Butler A, Hoffman P, Smibert P, Papalexi E, Satija R. Integrating single-cell transcriptomic data across different conditions, technologies, and species. *Nat Biotechnol*. 2018;36(5):411-420. doi:10.1038/nbt.4096

4. Wolf FA, Angerer P, Theis FJ. SCANPY: large-scale single-cell gene expression data analysis. *Genome Biol*. 2018;19(1):15. doi:10.1186/s13059-017-1382-0

5. Lopez R, Regier J, Cole MB, Jordan MI, Yosef N. Deep Generative Modeling for Single-cell Transcriptomics. *Nat methods*. 2018;15(12):1053-1058. doi:10.1038/s41592-018-0229-2

6. Ge SX, Jung D, Yao R. ShinyGO: a graphical gene-set enrichment tool for animals and plants. *Bioinformatics*. 2019;36(8):2628-2629. doi:10.1093/bioinformatics/btz931

7. Ashburner M, Ball CA, Blake JA, et al. Gene Ontology: tool for the unification of biology. *Nat Genet*. 2000;25(1):25-29. doi:10.1038/75556

8. Aleksander SA, Balhoff J, Carbon S, et al. The Gene Ontology knowledgebase in 2023. *Genetics*. 2023;224(1):iyad031. doi:10.1093/genetics/iyad031

9. Nishimura D. BioCarta. *Biotech Softw Internet Rep*. 2001;2(3):117-120. doi:10.1089/152791601750294344

10. Milacic M, Beavers D, Conley P, et al. The Reactome Pathway Knowledgebase 2024. *Nucleic Acids Res*. 2023;52(D1):D672-D678. doi:10.1093/nar/gkad1025

11. Thiriot A, Perdomo C, Cheng G, et al. Differential DARC/ACKR1 expression distinguishes venular from non-venular endothelial cells in murine tissues. *BMC Biol*. 2017;15(1):45. doi:10.1186/s12915-017-0381-7

12. Kitching AR, Holdsworth SR, Ploplis VA, et al. Plasminogen and plasminogen activators protect against renal injury in crescentic glomerulonephritis. *The Journal of experimental medicine*. 1997;185(5):963-968. http://eutils.ncbi.nlm.nih.gov/entrez/eutils/elink.fcgi?dbfrom=pubmed&id=9120402&retmode=ref&cmd=prlinks

13. Chen J, Chen JK, Conway EM, Harris RC. Survivin Mediates Renal Proximal Tubule Recovery from AKI. *J Am Soc Nephrol*. 2013;24(12):2023-2033. doi:10.1681/asn.2013010076

ctc gag ttt ttc agc aag **at ac atggcttttg aactgcct**tt ccttggatcc agttcaaggg gatggaggag
cagtgagagt cagccgccct tccactccaa tttcccagca cctcccttat ctctgcctca caagtcaccc agcccccctc
tcttccttcc ttgtgcttga agaactctcc cttgctggaa agccccctgt tttctcaatc tccctttcca cttcggtaaa
atctctactt gctggaaagc cccctgtttt ctcaatctcc ctttccactt cggtaaaatg cccactttct ggtccccacc
tttttcctga gtgtagtccc aaccagccaa atccaacctc aaaacaggaa gacccaaggc cagtgacccc
cataggcctg aggcttgtgc aggcagtggg cgtggggtaa ggcttcctga tgccccctgt ccctgcccag
aacctgatgg ccctcattag tccttggctc TTAC/TCT tgga agcacaggcg ctgacagccg tcccagccct
tctgtctgcg ggcctgaacc aaacggtgcc atggggaact gtctgcacag ggtgagtatg gggccaggcc
ccagagtccc ttatccctat gcccctcatt tcccgtgctg tttgcccctc agtctttata tctcttcctt ttcctcctca
tcttttctcc cttcctgctt ttttcctctt ccttcaaagt ctttttcctt ctctccttcc tatgctagcc tcctagctcc
ctcttgtgtc cctccctttg cctttgagtc agttccatcc tggtctcttg gtgccttttc cttctgacct tgcactgctc
ctccagcccc agctgccct ggcttccccag gactgttcct gctccggctc ttcaggctcc ctgctttgtc cttttccact
gtccgcactg catctgactc ctgcagagac cttgttctcc cacccgacct tcctctctgt cctcccctcc cacctgcccc
tcaattccca ggagactctt ccggtgtaac tctgatggcc tcctctgggt atgtcctcca ggcggagctc
tccccctcaa ctgagaactc aagtcagctg gacttcgaag atgtatggaa ttcttcctat ggtgtgaatg attccttccc
agatggagac tatgatgcca acctggaagc agctgccccc tgccactcct gtaacctgct ggatgactct
gcactgccct tcttcatcct caccagtgtc ctgggtatcc tagctagcag cactgtcctc ttcatgcttt tcagacctct
cttccgctgg cagctctgcc ctggctggcc tgtcctggca cagctggctg tgggcagtgc cctcttcagc attgtggtgc
ccgtcttggc cccagggcta ggtagcactc gcagctctgc cctgtgtagc ctgggctact gtgtctggta tggctcagcc
tttgcccagg ctttgctgct agggtgccat gcctccctgg ggcacagact gggtgcaggc caggtcccag
gcctcaccct ggggctcact gtgggaattt ggggagtggc tgccctactg acactgcctg tcaccctggc
cagtggtgct tctggtggac tctgcaccct gatatacagc acggagctga aggctttgca ggccacacac
actgtagcct gtcttgccat ctttgtcttg ttgccattgg gtttgtttgg agccaagggg ctgaagaagg cattgggtat
ggggccaggc ccctggatga atatcctgtg ggcctggttt attttctggt ggcctcatgg ggtggttcta ggactggatt
tcctggtgag gtccaagctg ttgctgttgt caacatgtct ggcccagcag gctctggacc tgctgctgaa cctggcagaa
gccctggcaa ttttgcactg tgtggctacg cccctgctcc tcgccctatt ctgccaccag gccacccgca ccctcttgcc
ctctctgccc ctccctgaag gatggtcttc tcatctggac acccttggaa gcaaatccta gttctcttcc cacctgtcaa
cctgaattaa agtctacact gcctttgtga agcgggtggt ttcttatttt gtctggggag aagaaggaga
atggagagag agacattttt atgtcagact ttcttgccag tgtctgcttc tatagctggc ttgggaagaa ggtgaatgat
gaataaatac cctcagggta cacagatgtt ctcttgaggt gtggggtcac ggccatctca agggagaaga
gaagaggaac cagagcatga ggggagtcat taaaccaaaa aaaacagaag ggatggctta gctggaaaaa
aagctgttct gggaagcaaa tggaatagga actcaaactg agagataaac agtgaagagt gatgacaaag
cccagagcaa taccacctcc ccctgtccaa cctgcccagc ctctgtcttc tgtctcctct ctggctttgt ttagtgatta
ggacagtggt ggggaaggtg aaagaagcat cccaggggat gttactcagt tcagggaaca tatcaaggta
atttaaaaag ccacttcctg ggagtcatct ctcccaggtt cctcagcatg acctgaatgt gcgtgc gtgt gtgtgtgtgt
gtgtgtgtac acatctgttt ctcgatctgttagaatctac ctttatgtta gatgtatgca tgtaaaaaca tatgtccacc
catgagcttg catctctgtc agcacctgaa ctgcgcacac ctgtgcgtgt gcactgactt ttctcaggac ccaaaccccc
actcaattct gcactcatcc ctgttcacag gatatagaat cgggatttat gactcactcc ttacccaaat gagttttctt
taccctggtt tttaagccta gtcttttctg tgtaggatgt gtggagggaa gaaaagatca agaagttgtg aagggtggag
aaacttgaag ggggaggccc tgatttgatt catcttctgc ttggaattcc ccgaatttcc ctttcagaat ctcagctttt
gaaataaacc tttatttccc acatacatct ttccttccac cttccacaca ataccccaat cccctgggca cctttttccc
aacccctgat tctctggctg cttaatcatg acctttgaga tttttctcag tctctaccta cccaagttta gatggctgga
aggacagaaa cccctcctca tcaggggcac agcttttacc accaaga **gca aattcaccct ctacccaaga**
ggatctttct aga

Supplemental Figure 1 – Map of cloned construct for the generation of transgenic humanized mice

Exon Sequence: grey highlight; Intron: *cursive font;* GATA-1 site: CAPITAL LETTERS with red highlight WT/MUT; FY*B: blue highlight; Primer sequence used for isolation: bold font; Vector sequence: blue font. WT, wildtype; MUT, mutant.

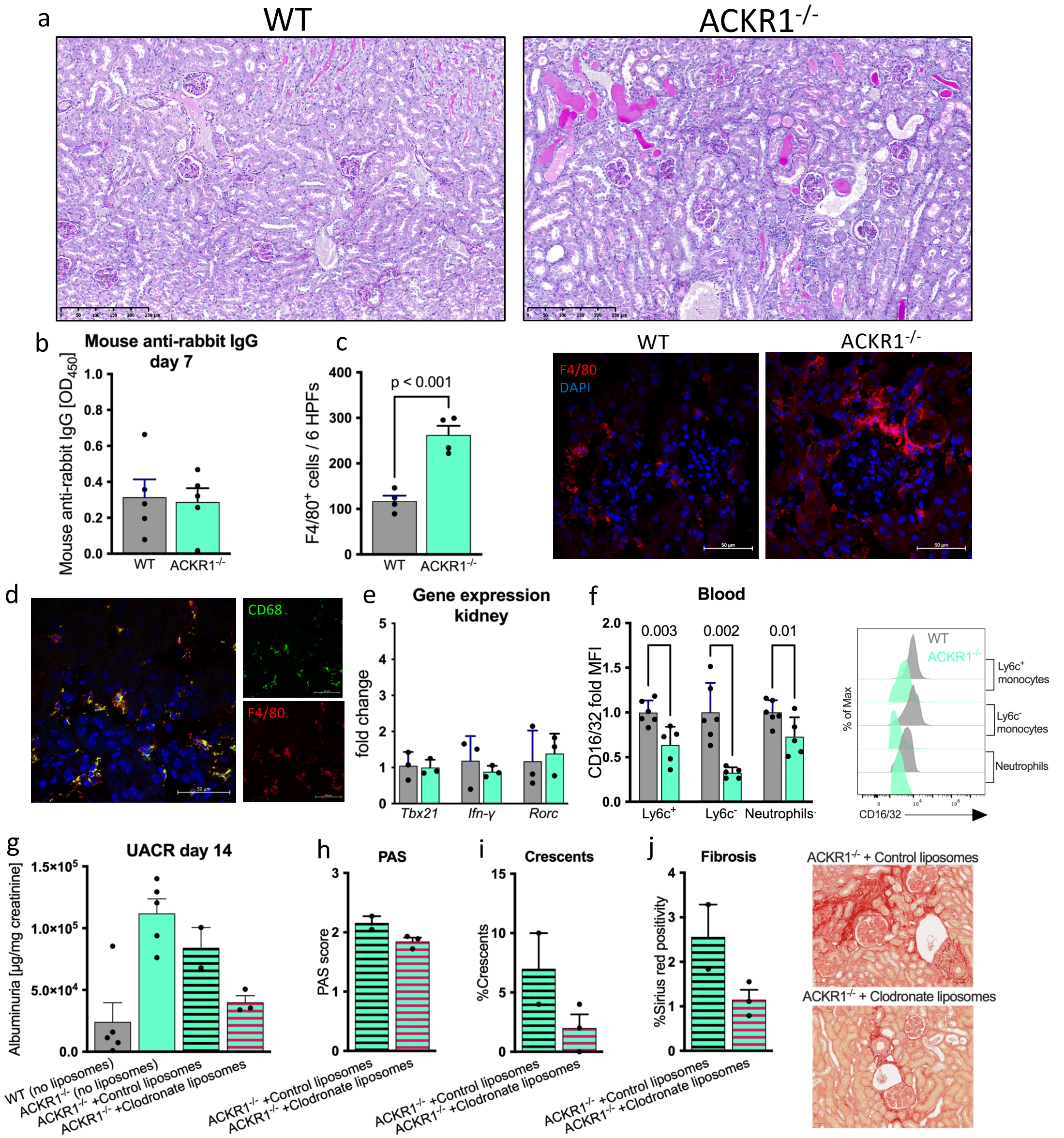

Supplemental Figure 2– Disease phenotype in nephritic WT and *Ackr1*$^{-/-}$ mice.

(**a**) Low-magnification periodic acid-Schiff stained kidney sections from WT and *Ackr1*$^{-/-}$ mice 14 days after induction of nephrotoxic serum nephritis. (**b**) 1:64000 dilution of mouse anti-rabbit IgG in nephritic WT and *Ackr1*$^{-/-}$ mice 7 days after induction of nephrotoxic serum nephritis measured ad OD450. (**c**) Increased F4/80^{+} cells in kidneys of *Ackr1*$^{-/-}$ mice as compared to WT mice. Representative micrographs are shown. (**d**) Expression of CD68 and F4/80 on renal macrophages. (**e**) Quantitative real-time PCR of total kidney tissue isolated from nephritic WT and *Ackr1*$^{-/-}$ mice. (**f**) Expression of CD16/32 on monocytes and neutrophils in the peripheral blood of nephritic WT and *Ackr1*$^{-/-}$ mice. (**g**) *Ackr1*$^{-/-}$ mice were treated with control or clodronate liposomes, and albuminuria was compared to untreated WT and *Ackr1*$^{-/-}$ mice 14 days after induction of NTSN. (**h**) PAS, (**i**) Crescents and (**j**) quantification of fibrosis and representative micrographs of Picro-Sirius red stainings in *Ackr1*$^{-/-}$ mice treated with control or clodronate liposomes. n=6 WT mice in (**f**); n=5 mice/group in (**b**) as well as WT mice in (**f**) and mice not treated with liposomes (**g**); n=4/group in (**c**); n=3 mice/group (**e**), mice treated with clodronate liposomes (**g-j**) and n=2 mice treated with control liposomes (**g-j**). Scale bars, 250µm (**a**), 50µm (**c,d,j**). All data show Mean+SEM. WT, wildtype.

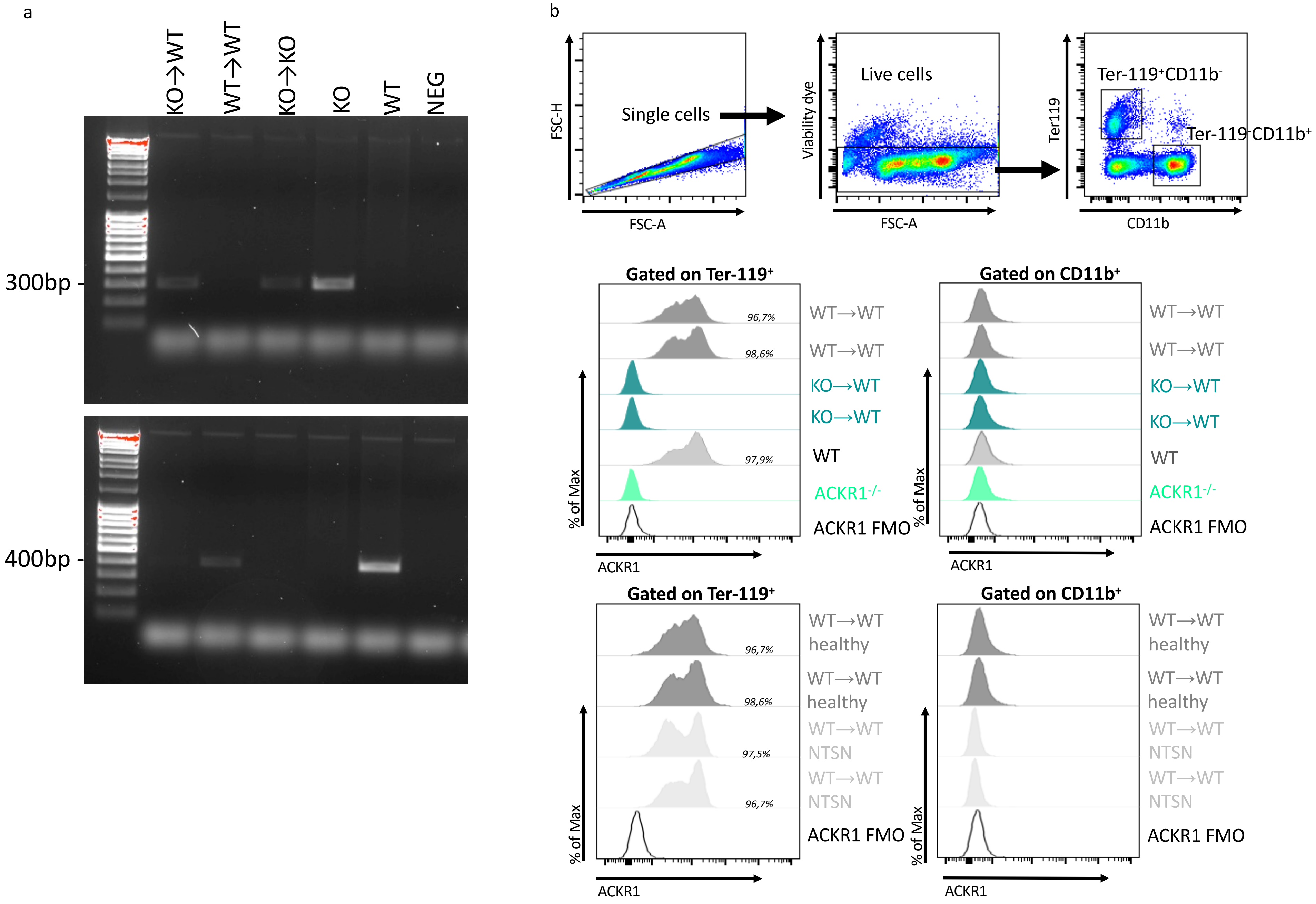

Supplemental Figure 3 - Successful bone marrow chimerism

Bone marrow chimerism was confirmed by (**a**) Amplification of a 300-bp fragment of the targeted *Ackr1* gene (upper gel), and a 400-bp fragment of the wildtype *Ackr1* allele (lower gel) and (**b**) flow cytometry. Gating strategy and representative histograms (ACKR1) of bone marrow from two irradiated WT mice reconstituted with WT or ACKR1$^{-/-}$ cells as well as WT, *Ackr1*$^{-/-}$, and fluorescence minus one (FMO) controls. Histograms gated on Ter-119^{+} and CD11b^{+} cells. WT, wildtype; KO, knock-out; NEG, negative control; FMO, fluorescence minus one control.

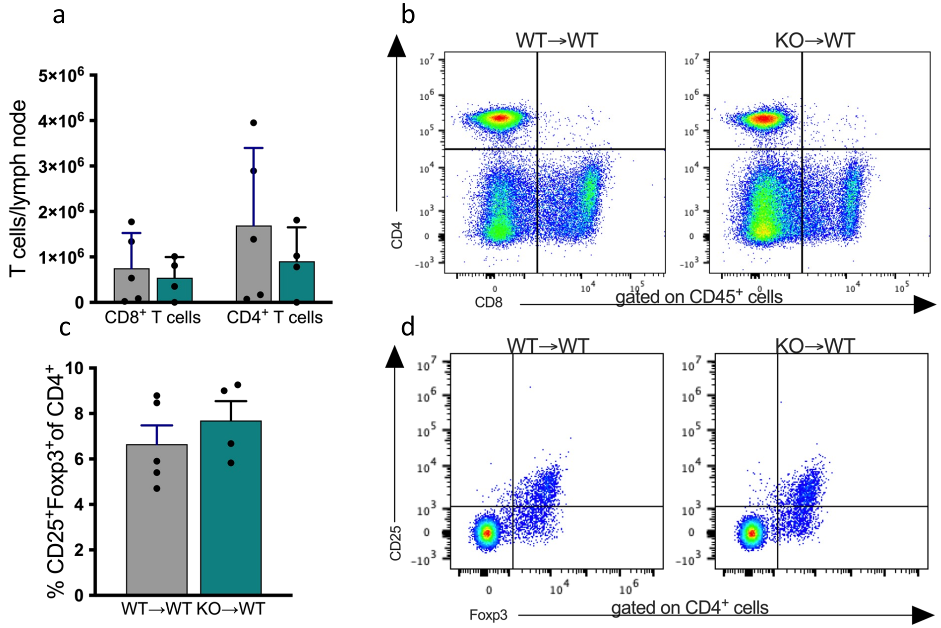

Supplemental Figure 4– Erythroid ACKR1 deletion does not impact T cell immunity and regulatory differentiation

(**a**) absolute numbers and (**b**) representative flow cytometry plot of $CD8^+$ and $CD4^+$ T cells in lymph nodes from irradiated WT mice reconstituted with bone marrow cells from WT or *Ackr1*$^{-/-}$ mice. (**c**) percentages and (**d**), representative flow cytometry plot of $CD4^+$ regulatory T cells from lymph nodes of WT mice reconstituted with bone marrow cells from WT or *Ackr1*$^{-/-}$ mice. n=5 in mice reconstituted with bone marrow cells from WT mice and n=4 in mice reconstituted with bone marrow cells from *Ackr1*$^{-/-}$ mice. All data show Mean+SEM. WT, wildtype; KO, knock-out.

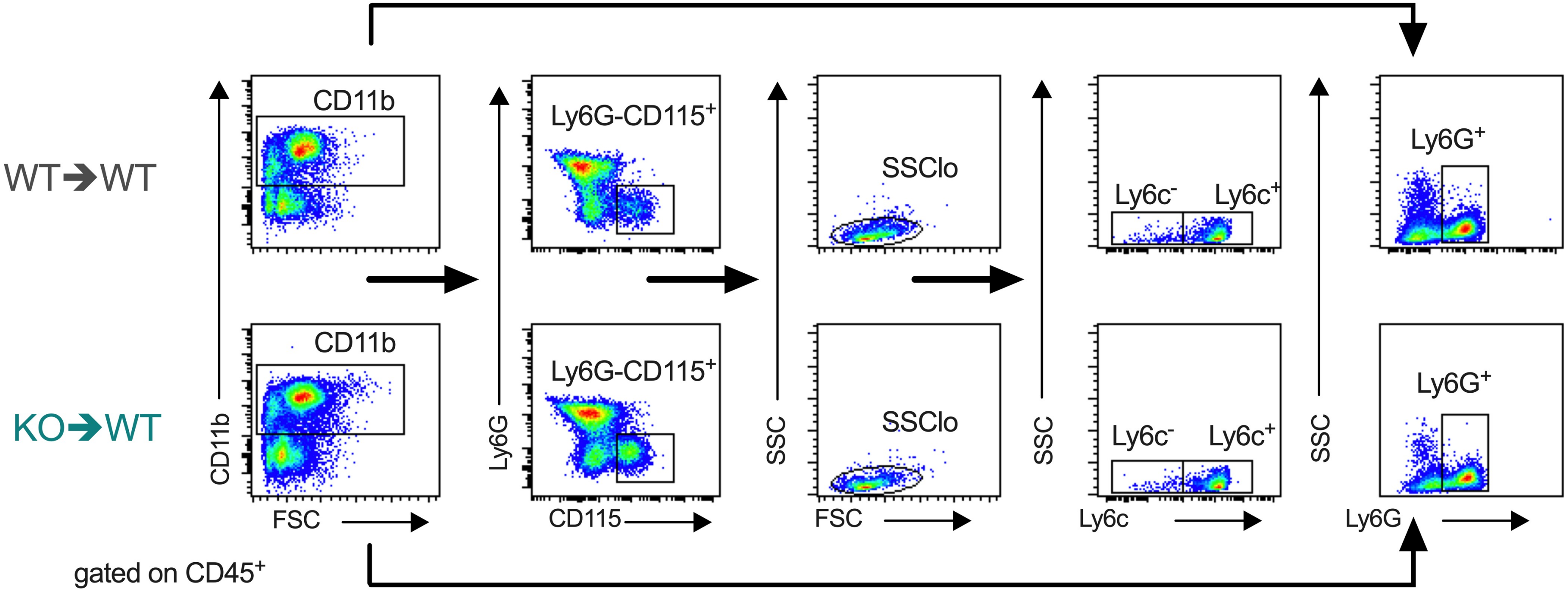

Supplemental Figure 5 – Gating strategy for classical and non-classical monocytes as well as neutrophils. Ly6c$^-$ and Ly6c$^+$ monocytes as well as Ly6G$^+$ neutrophils in bone marrow and kidneys as determined by flow cytometry of WT mice after transplantation with WT or *Ackr1*$^{-/-}$ cells. WT, wildtype, KO, knock-out.

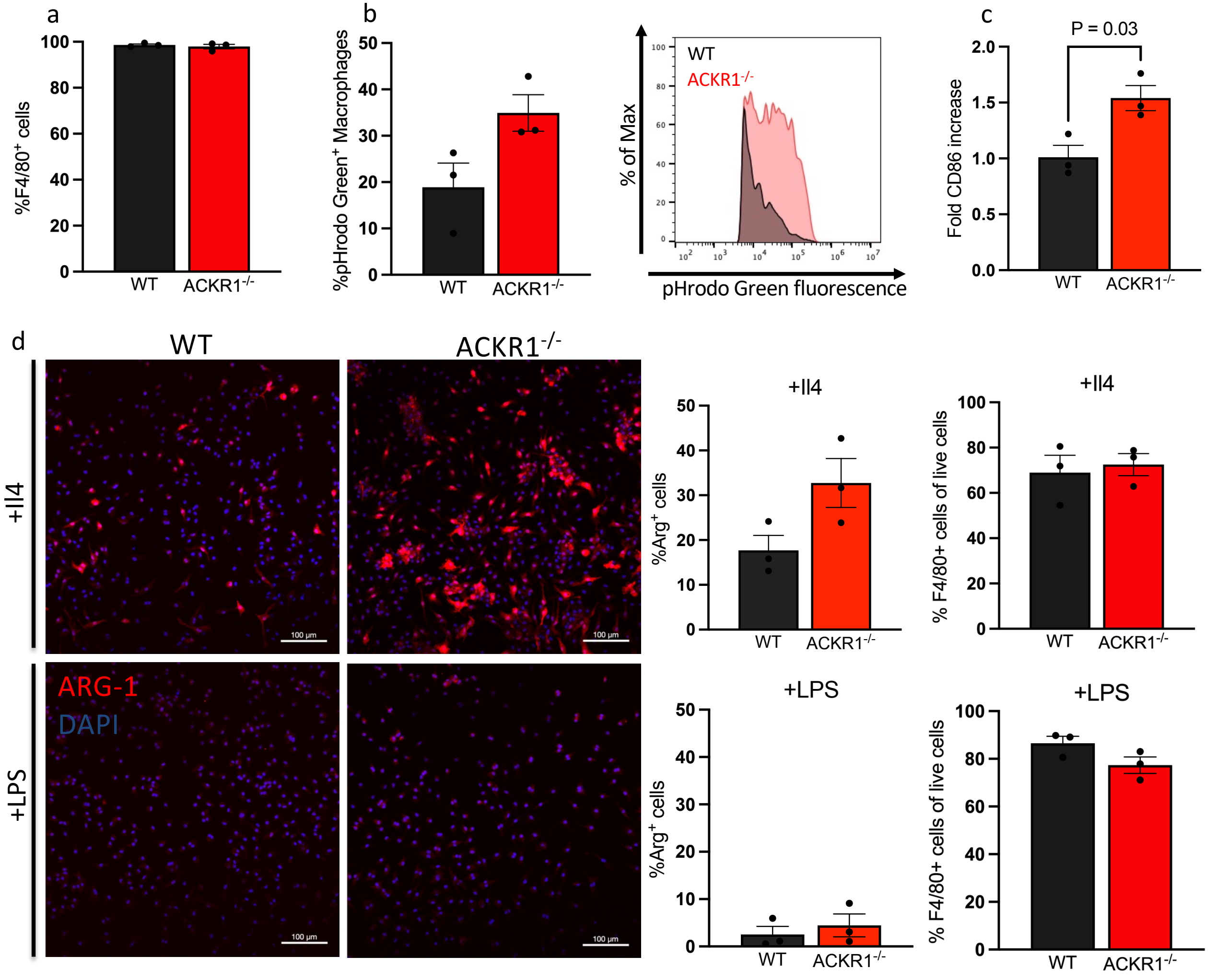

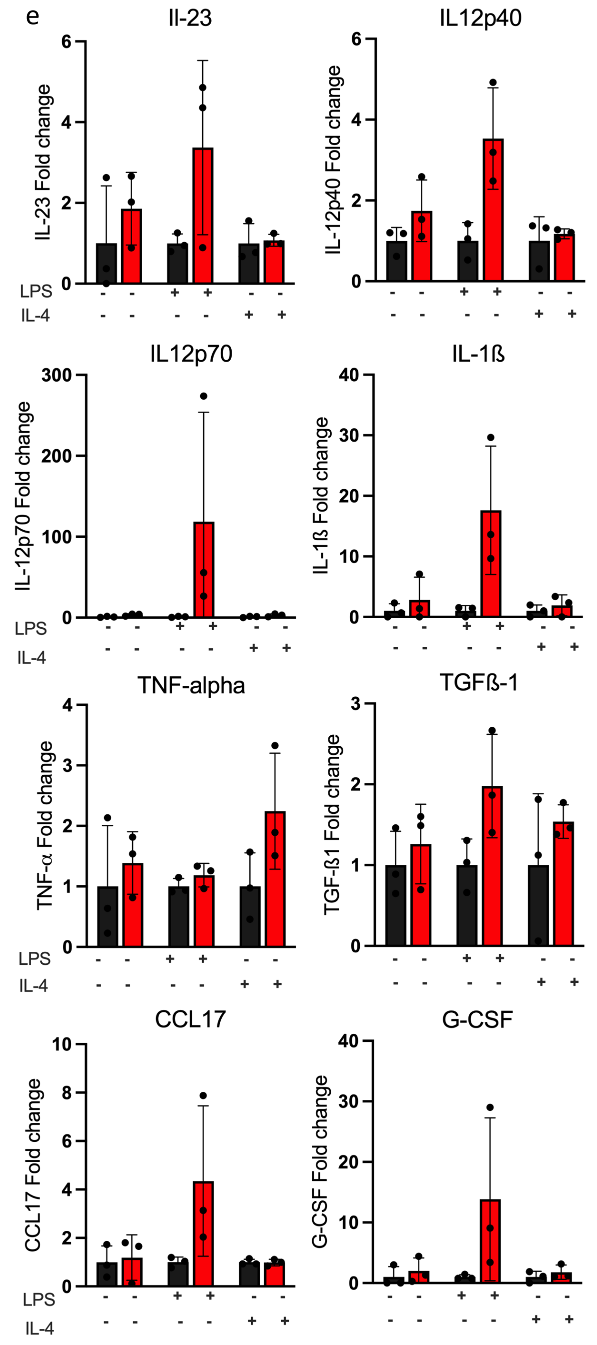

Supplemental Figure 6– ACKR1-deficient bone marrow-derived macrophages are hyperreactive

(**a**) Proportion of F4/80⁺ cells was assessed in bone marrow-derived macrophages following 7 days of in vitro culture, using quantitative analysis of immunofluorescence stainings (**b**) Percentage of S. aureus pHrodo Green⁺ bone marrow-derived macrophages and representative histograms of pHrodo Green fluorescence and (**c**) CD86 mRNA expression in bone marrow-derived macrophages from WT and ACKR1$^{-/-}$ mice. (**d**) Arg-1 and F4/80 expression on bone marrow-derived macrophages from WT and *Ackr1*$^{-/-}$ mice polarized with Il4 or LPS. Quantification of Arg-1⁺ cells by immunofluorescence stainings and analysis of F4/80 expression by flow cytometry in bone marrow-derived macrophages. (**e**) Cytokines in the supernatant of bone marrow-derived macrophages polarized with Il-4 or LPS**.** n=3 mice/group. Scale bars, 100µm. All data are given as mean±SEM. WT, wildtype; LPS, lipopolysaccharide; Arg-1, Arginase 1.

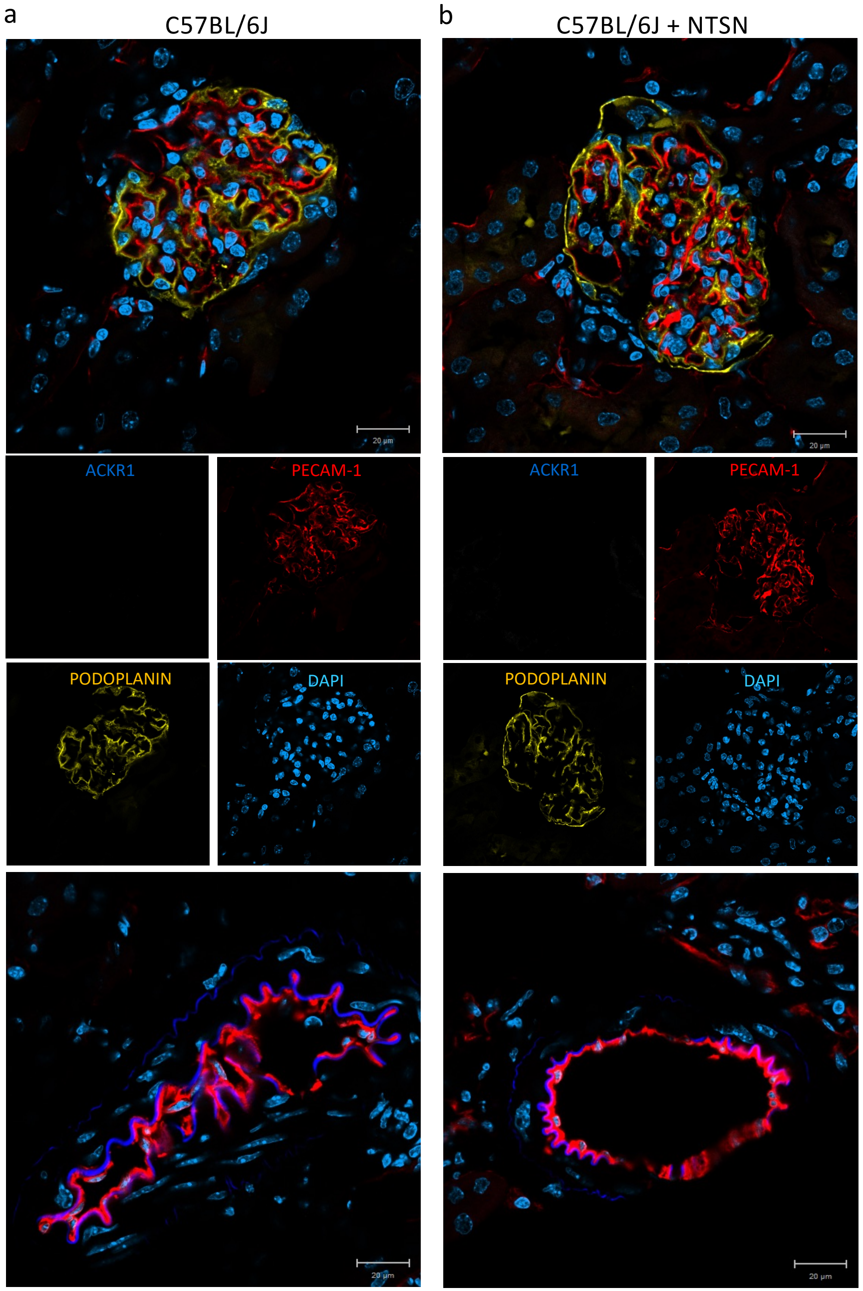

Supplemental Figure 7 - ACKR1 is not expressed in murine glomeruli Immunofluorescence micrographs of (**a**), healthy C57BL/6J kidney and (**b**), C57BL/6J kidney after 14 days of nephrotoxic serum nephritis stained with anti-ACKR1 (dark blue), anti-PECAM-1 (endothelial cells, red), Podoplanin (podocytes, yellow) and DAPI (nuclei, blue). Micrographs show glomeruli (upper panel) and renal blood vessels (lower panel). Scale bars, 20μm. NTSN, nephrotoxic serum nephritis.

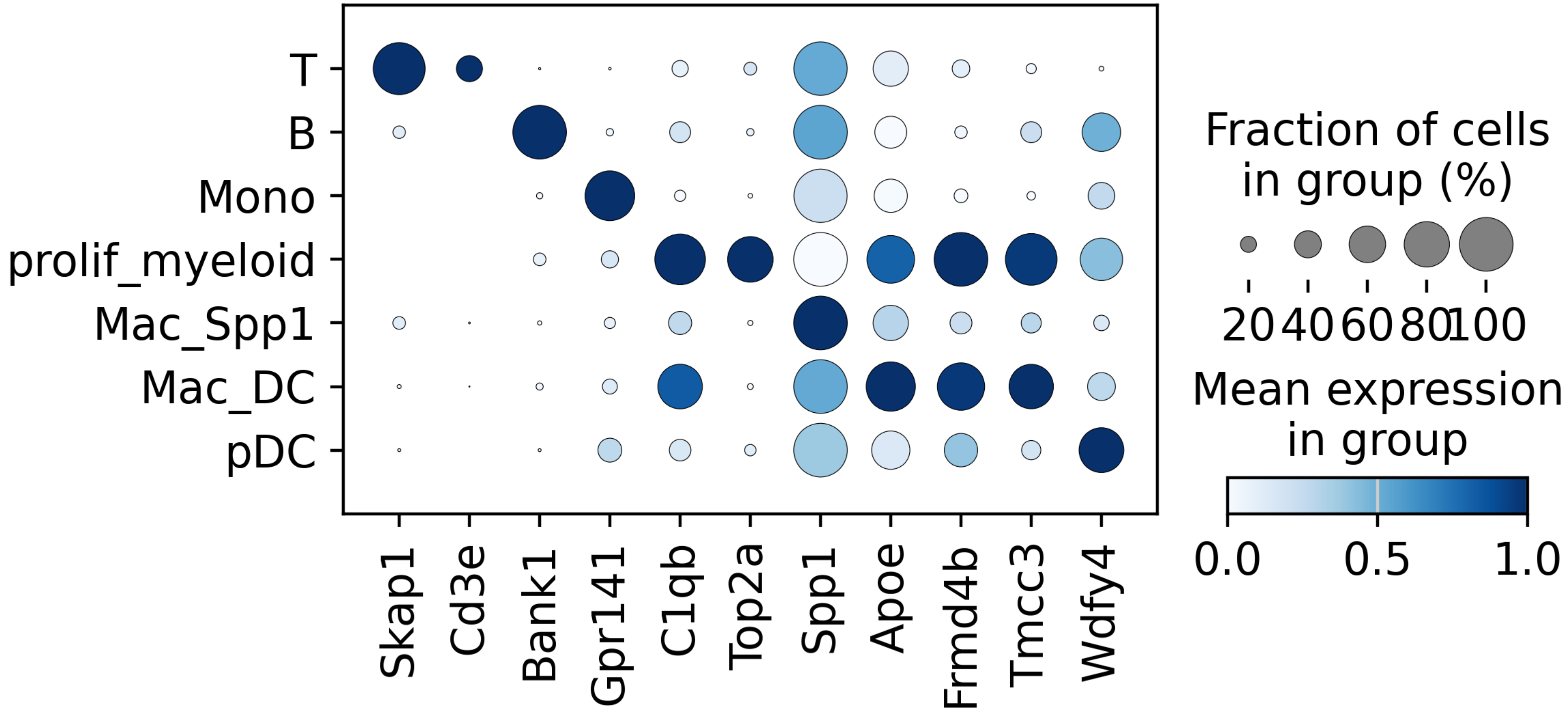

Supplemental Figure 8 – Immune subclustering marker genes
Dot plot of marker genes in identified immune cell populations.

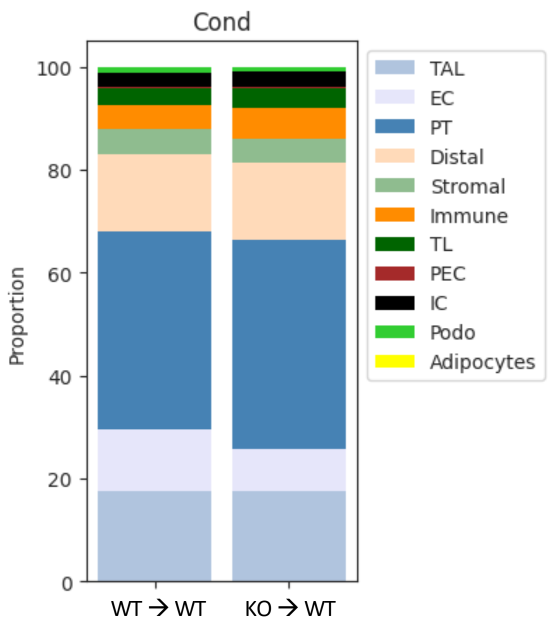

Supplemental Figure 9 – Relative proportions of major cell populations
snRNAseq analysis revealed comparable relative proportions of all major cell populations, except for increased proportions of immune cells.

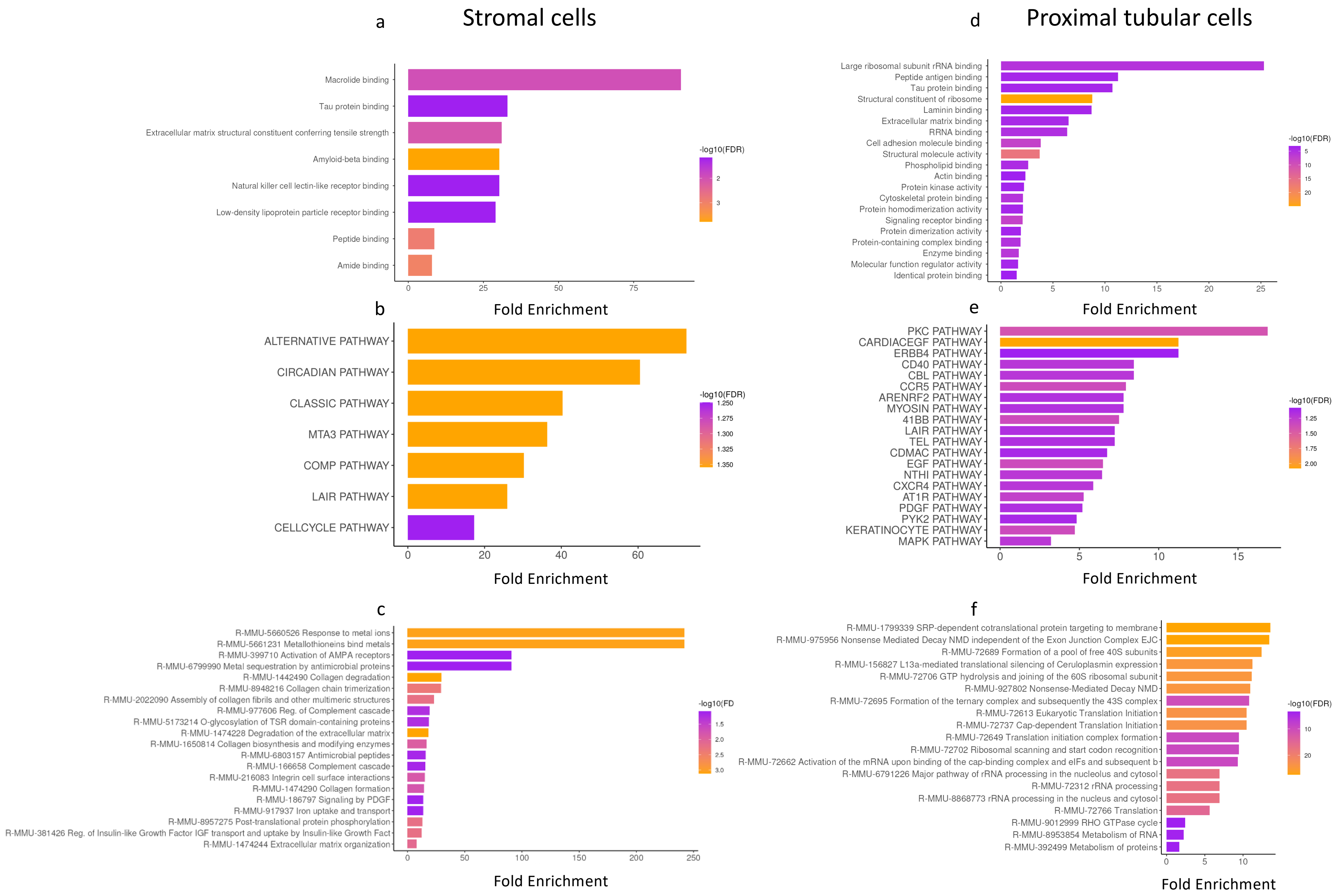

Supplemental Figure 10 – Functional pathway analysis in stromal and proximal tubular cells
Pathway enrichment analysis of differentially expressed genes in stromal cells (**a-c**) and proximal tubular cells (**d-f**) in WT→WT and KO→WT. Results from (**a+d**) gene ontology analysis molecular function, (**b+e**) Biocarta, and (**c+f**) Reactome databases were analyzed using ShinyGO 0.82. For each database, up to 20 pathways (FDR<0.1) are shown. Pathway enrichment analysis of upregulated genes was performed separately for each database and cell type.
.

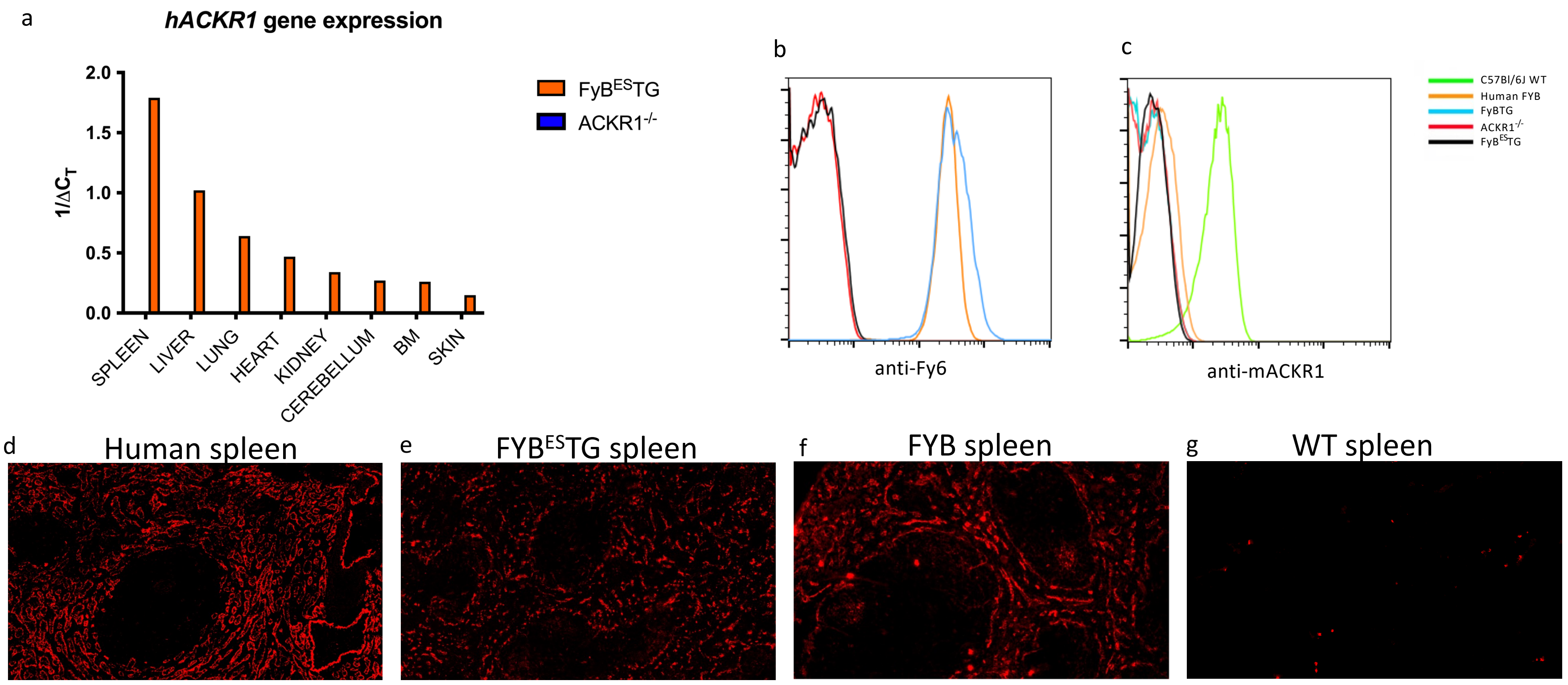

Supplemental Figure 11 – Erythrocyte ACKR1 expression in ACKR1$^{-/-}$, FYB^{ES}TG and respective controls

(**a**), ACKR1 gene expression in humanized ACKR1 FYB^{ES}TG and genotype negative control tissues. (**b+c**), Erythrocyte expression of human ACKR1 in (**b**), FYB^{ES}TG and FYBTG mice with anti-Fy6 antibody, with *Ackr1$^{-/-}$* and human ACKR1 positive controls. (**c**) Erythroid cell antibody staining confirmed the absence of murine ACKR1. Graphs representative of erythroid ACKR1 expression in at least 7 animals of respective strains. (**d-g**), anti-Fy6 Immunofluorescence staining of (**d**), human spleen, (**e**), FYB^{ES}TG spleen, (**f**) FYB spleen and (**g**), WT spleen. Magnification, x10 (**f,g**), Magnification, x25 tile scan (**d**), Magnification, x40 tile scan (**e**). WT, wildtype; WT, wild-type; FyBTG, FyB transgene; FyB^{ES}TG, FyB^{ES} transgene. BM, bone marrow.

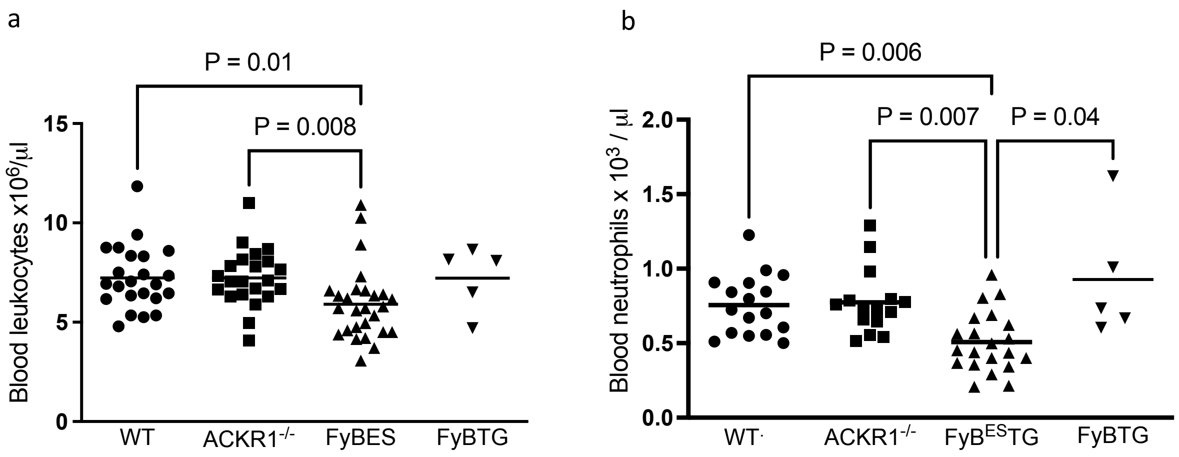

Supplemental Figure 12 – Differential blood leukocyte and blood neutrophil count

Peripheral (**a**), blood leukocyte count in the blood of WT (n=23), ACKR1$^{-/-}$ (n=22), FYB^{ES}TG (n=27) and FYBTG (n=5). and (**b**), neutrophil count in the blood of WT (n=17), *Ackr1$^{-/-}$* (n=16), FYB^{ES}TG (n=21) and FYBTG (n=5). WT, wild-type. FyBTG, FyB transgene; FyB^{ES}TG, FyB^{ES} transgene.

Group	Deposition of mouse anti-rabbit IgG (titer)
**WT**	1:1600
**ACKR1$^{-/-}$**	1:1600
**FyBTG**	1:1600
**FyB^{ES}TG**	1:1600

Supplemental Table 1 – Autologous antibody deposition in glomeruli of nephritic mice.
Mouse anti-rabbit IgG was measured by determining titers of immunofluorescence positivity in glomeruli. N=9/group in WT and *Ackr1*$^{-/-}$ mice and n=7/group in FyBTG and FyBES. WT, wild-type; FyBTG, FyB transgene; FyB^{ES}TG, FyB^{ES} transgene.

Supplemental Table 2 – Gene expression of myeloid cells, stromal cells and proximal tubular cells in chimeric WT mice reconstituted with WT cells and ACKR1$^{-/-}$ cells.

Cell types shown are myeloid cells, stromal cells and proximal tubular cells in snRNA-seq.
